# High-Affinity Inhibitors of Human NAD^+^-Dependent 15-Hydroxyprostaglandin Dehydrogenase: Mechanisms of Inhibition and Structure-Activity Relationships

**DOI:** 10.1371/journal.pone.0013719

**Published:** 2010-11-02

**Authors:** Frank H. Niesen, Lena Schultz, Ajit Jadhav, Chitra Bhatia, Kunde Guo, David J. Maloney, Ewa S. Pilka, Minghua Wang, Udo Oppermann, Tom D. Heightman, Anton Simeonov

**Affiliations:** 1 Structural Genomics Consortium, Nuffield Department of Clinical Medicine, University of Oxford, Oxford, United Kingdom; 2 NIH Chemical Genomics Center, National Human Genome Research Institute, National Institutes of Health, Bethesda, Maryland, United States of America; 3 Biomedical Research Unit, Nuffield Department of Orthopedic Surgery, Rheumatology and Musculoskeletal Sciences, Botnar Research Center, University of Oxford, Oxford, United Kingdom; University of Cambridge, United Kingdom

## Abstract

**Background:**

15-hydroxyprostaglandin dehydrogenase (15-PGDH, EC 1.1.1.141) is the key enzyme for the inactivation of prostaglandins, regulating processes such as inflammation or proliferation. The anabolic pathways of prostaglandins, especially with respect to regulation of the cyclooxygenase (COX) enzymes have been studied in detail; however, little is known about downstream events including functional interaction of prostaglandin-processing and -metabolizing enzymes. High-affinity probes for 15-PGDH will, therefore, represent important tools for further studies.

**Principal Findings:**

To identify novel high-affinity inhibitors of 15-PGDH we performed a quantitative high-throughput screen (qHTS) by testing >160 thousand compounds in a concentration-response format and identified compounds that act as noncompetitive inhibitors as well as a competitive inhibitor, with nanomolar affinity. Both types of inhibitors caused strong thermal stabilization of the enzyme, with cofactor dependencies correlating with their mechanism of action. We solved the structure of human 15-PGDH and explored the binding modes of the inhibitors to the enzyme *in silico*. We found binding modes that are consistent with the observed mechanisms of action.

**Conclusions:**

Low cross-reactivity in screens of over 320 targets, including three other human dehydrogenases/reductases, suggest selectivity of the present inhibitors for 15-PGDH. The high potencies and different mechanisms of action of these chemotypes make them a useful set of complementary chemical probes for functional studies of prostaglandin-signaling pathways.

**Enhanced version:**

**This article can also be viewed as an enhanced version in which the text of the article is integrated with interactive 3D representations and animated transitions. Please note that a web plugin is required to access this enhanced functionality. Instructions for the installation and use of the web plugin are available in Text S2.**

## Introduction

Eicosanoids are arachidonic acid derivatives that comprise distinct functional classes including prostaglandins (PGs), lipoxins and leukotrienes [1]–[3]. These bioactive fatty acids control a multitude of physiological functions including inflammation and differentiation. Dysregulation of the enzymes responsible for the generation and metabolism of active prostaglandins and lipoxins is associated with malignant transformation and progression in a variety of cancer types, such as breast, colon, lung and bladder cancers [4]–[10]. The intracellular levels of prostaglandins are controlled mainly by the interplay between the cyclooxygenase enzymes (COX-1 and COX-2) on the one hand, and 15-hydroxyprostaglandin dehydrogenase (15-PGDH, EC 1.1.1.141) and other inactivating enzymes, on the other. COX-1 and COX-2 are bifunctional enzymes that, through their fatty acid cyclooxygenase- and prostaglandin hydroxyperoxidase activities, ultimately catalyze the generation of prostaglandin H_2_ (PGH_2_) from arachidonic acid [11]. The other prostaglandins are then generated and transformed into one another by several isomerases and synthases, producing, e.g., prostacyclin (PGI_2_) or thromboxane A_2_ (TX) [11], [12]. The wide variety of effects of prostaglandins in many different cell types derives from binding to a variety of mainly G-protein coupled receptors, of which eleven are currently known [12]. In addition, prostaglandins also interact with nuclear hormone receptors, thereby eliciting direct transcriptional effects [13], [14].

15-PGDH represents the key enzyme in the inactivation of a number of active prostaglandins, leukotrienes and hydroxyeicosatetraenoic acids (HETEs) (e.g., by catalyzing oxidation of PGE_2_ to 15-keto-prostaglandin E_2_, (15k-PGE)) [15]. Thus far, two forms of 15-PGDH have been identified: NAD^+^-dependent type-I 15-PGDH, and the NADP^+^-dependent type-II 15-PGDH [15], also known as carbonyl reductase 1 (CBR1, SDR21C1) [16], [17]. The preference for NADP^+^ and the high *K*_m_ values of CBR1 for most PGs suggest that the majority of the *in vivo* activity can be attributed to type-I 15-PGDH [15]. Human type-I 15-PGDH is encoded by the *HPGD* gene and belongs to the evolutionarily conserved superfamily of short-chain dehydrogenase/reductase enzymes (SDRs) [18], within which it is classified as SDR36C1 [17]. The enzyme has been purified from human placenta and its primary structure determined by Edman degradation [19]; it was subsequently cloned [20] and characterized as a homodimer with subunits of a size of 29 kDa [20], [21].

The critical importance of 15-PGDH for the inactivation of prostaglandins makes the enzyme an attractive target for studying the details of interactions and signaling events in inflammation and cancer. However, all inhibitors that have been identified so far lack potency and specificity. Several thiazolidinedione peroxisome proliferator-activated receptor γ (PPARγ) agonists, including pioglitazone and ciglitazone, have been shown to inhibit recombinant human placental 15-PGDH. Ciglitazone showed an IC_50_ of 2.7 µM [22], while an optimized derivative, CT-8, had a *K*_i_ of ∼90 nM [23]. Other clinically approved drugs that also act as inhibitors of 15-PGDH with micromolar potencies include non-steroidal anti-inflammatory drugs (NSAIDs) and COX inhibitors, e.g., indomethacin, sulindac, and niflumic acid [22]. Finally, a group of compounds called sulphasalazines have also been shown to inhibit human 15-PGDH, with the most effective compound, CAY10397, having a *K*_i_ of 110 nM [24].

Here we describe the identification of chemotypes that potently inhibit human 15-PGDH, with either competitive or noncompetitive kinetics, and strongly stabilize the enzyme in a cofactor-dependent manner. The lead compounds show remarkable selectivity based on accumulated data from a large number of high-throughput screens against a wide range of targets. The determination of the crystal structure of 15-PGDH, also reported in this work, enabled us to propose a binding mode for the competitive inhibitor in the active site pocket that confirms its mechanism of inhibition. For the other inhibitors, the observations from the 15-PGDH crystal structure characterized the mechanism of inhibition as being noncompetitive.

## Results

### High-throughput identification of inhibitors of 15-PGDH

**Figure 1:**
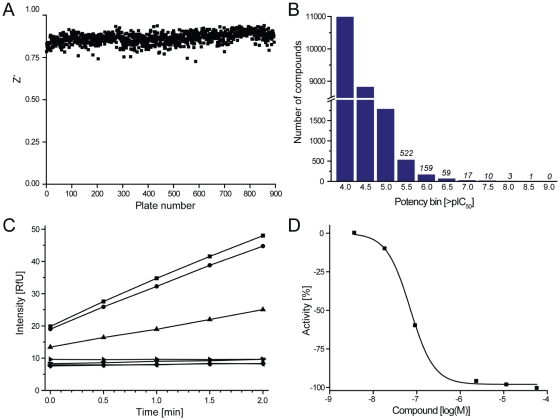
Quantitative high-throughput screen of 15-PGDH. *A.* Plot of the Z' factor associated with each plate, showing high stability over the entire duration of the screen (completed in five days). The average Z' was 0.86. *B.* Hit frequency for the library of tested compounds, measured as the distribution of compounds according to binned potencies. *C.* Typical effect of a non-fluorescent screening hit (inhibitor **13**, titrated between 3.5 nM and 57.5 µM) on the time course of NAD^+^-reduction upon addition of PGE_2_. *D.* Dose-dependent reduction in enzyme activity caused by compound **13**, as detected during the screen.

To screen for inhibitors in a high-throughput format, we adopted the standard assay used for this enzyme which involved monitoring the increase in sample fluorescence corresponding to the conversion of the non-fluorescent NAD^+^ cofactor into the fluorescent NADH upon oxidation of the 15-PGDH substrate 15-hydroxyprostaglandin type-E_2_ (PGE_2_) (Figure 1). The assay was miniaturized to a 4-µL volume in 1536-well format: enzyme (3 µL) was dispensed first, followed by a pintool transfer of library compounds dissolved in DMSO. After equilibration, a substrate dispense (1 µL) initiated the enzymatic reaction (see Materials and Methods, for detailed protocol description). A robotic validation run consisting of a concentration-response screen of the LOPAC^1280^ library, performed in triplicate, yielded excellent assay statistics and hit reproducibility (supplementary information Figures S1A and S1B). The concentration-response screen of the entire collection comprising 895 1536-well plates was completed in 5 days. The Z' screening factor [25] associated with each plate remained high and stable throughout the screen (average Z' = 0.86, Figure 1A). A concentration response of the control inhibitor GW5074, identified in an earlier pilot screen (PubChem AID 894, supplementary information Figure S1C), added as a 16-point dilution series in duplicate between 57.5 µM and 3.5 nM into the second column of every assay plate displayed consistent inhibition throughout the screen (supplementary information Figure S1D): the average IC_50_ was 10.4 µM and the associated minimum significant ratio was 1.5, indicating a highly stable run according to the definition given by Eastwood and co-workers [26]. The hits identified ranged in inhibitory potency from submicromolar to double-digit micromolar (Figure 1B). In order to progress only the highest-confidence primary screening hits to the subsequent investigations, samples exhibiting weak/noisy responses, as well as those representing potential false positives, were eliminated during the initial analysis. The testing of each library compound in dose-response mode (see example in Figures 1C and 1D) permitted a detailed examination of the type and quality of the inhibition response (IC_50_, concentration-response curve shape, efficacy, presence of asymptotes). Hits associated with low-efficacy (<40% maximum inhibition), incomplete curves, as well as those displaying only a single-point inhibition at the top concentration tested were eliminated from further consideration. Similarly, the collection of real-time kinetic data allowed the autofluorescence of each compound to be evaluated by comparing the initial fluorescence read at top concentration with the mean value of uninhibited assay controls: compounds whose top-concentration initial well fluorescence exceeded six standard deviations over the mean were flagged as potential autofluorescent artifacts [27], [28] (see example in supplementary information Figure S1E). The complete screening and followup results are available in PubChem (PubChem Assay ID 894).

### Re-testing of HTS hits

**Figure 2:**
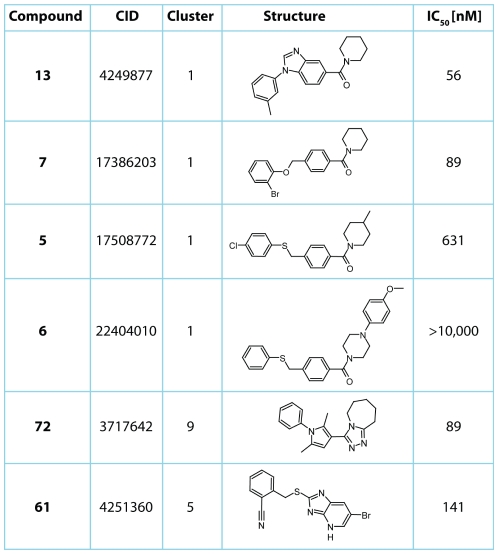
IC_50_ from re-testing for selected inhibitors of human 15-PGDH identified in the quantitative high-throughput screen. Shown are the IC_50_ values for select compounds arranged by cluster number, along with their PubChem Chemical Identifiers (CID).

Similarity clustering of the high-confidence compounds resulting from the above triaging process performed using LEADSCOPE (Columbus, OH, USA) yielded 23 clusters and 15 singletons. A total of 87 representative members were chosen for re-sourcing and re-testing in a miniaturized screening assay as 24-point dilution series [29] where the majority of the compounds confirmed. Visual inspection of these retested hits (supplementary information Table S1, a subset shown in Figure 2) allowed further merging of clusters. Based on these clusters, 50 representative compounds that fulfilled stringent criteria for confidence (i.e., complete concentration-response curves comprising two clear asymptotes, ≥80% max. inhibition, and R^2^>0.9) were selected for further evaluation in the protein stabilization experiments described below.

### Compound stabilization of 15-PGDH

**Figure 3:**
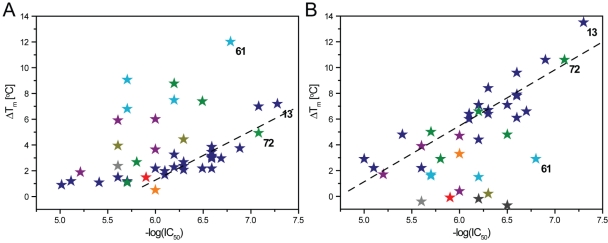
Correlation between thermal stabilization of 15-PGDH in presence of NAD^+^ (*A*) or NADH (*B*) and inhibitory pIC_50_ for screening hits from different structural clusters. Thermal stabilization is expressed as a shift in the midpoint of the unfolding transition of the protein (ΔT_m_). ΔT_m_ and pIC_50_ (-log(IC_50_) values are means of at least three independent measurements. The inhibitors are represented by color-coded symbols according to their chemical clusters, respectively (supplementary information Table S1): blue, cluster 1; dark green, cluster 2; orange, cluster 3; purple, cluster 4; light blue, cluster 5; light green, cluster 8; grey, cluster 9; dark yellow, cluster 11; red, singletons (a T_m_ could not be determined for the tested compounds in clusters 6, 7 & 10). The dotted line in each graph *A* & *B* denotes correlations for the compounds in cluster 1, respectively. Numbers within the graphs assign the positions for the inhibitors of special interest (see text).

**Figure 4:**
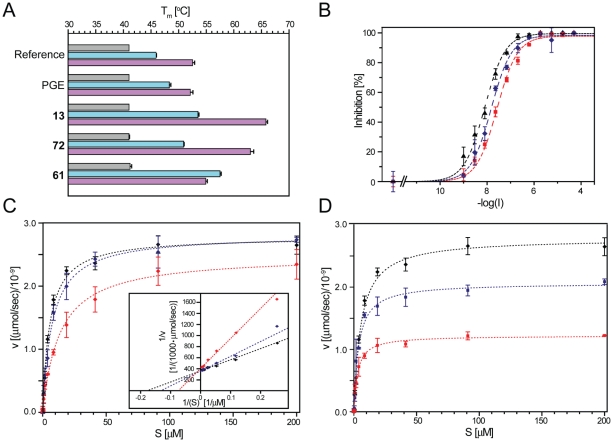
Effects of the re-synthesized compounds on stability and activity of human 15-PGDH. *A.* Thermal stability of the protein in dependence of substrate and inhibitors, without cofactor (grey bars), and in presence of NAD^+^ (light blue bars) as well as in presence of NADH (purple bars). Shown are sets of these data, respectively, for each of the tested compounds as denoted on the Y axis. Bars represent averages of three independent experiments, with standard deviations displayed as error bars. *B.* Inhibitor dose-dependent reduction of the enzyme activity caused by compounds **13** (black triangles), **72** (blue squares) and **61** (red diamonds), respectively. Plotted values are means of three independent experiments, with standard deviations displayed as vertical error bars. Dashed or dotted lines, in colors corresponding to the data points, result from least-squares non-linear fitting of the data to the Hill equation (see Materials & Methods), respectively. *C & D.* Michaelis-Menten plots of the enzyme activity at varying concentrations of substrate, PGE_2_, in absence of inhibitor (black symbols) or in presence of compounds **61** (*C*) or **13** (*D*) at concentrations close to (blue symbols) or above (red symbols) their IC_50_ values, specified in table 3. The inset graph in *D* shows the data from the main graph as Lineweaver-Burk plot, demonstrating competitive inhibition from independence of the Y-axis intercept (1/*V*_max_) of the inhibitor concentration. All values are means of three independent measurements; standard deviations are displayed as vertical error bars.

To investigate the ability of the inhibitors to thermally stabilize 15-PGDH, differential scanning fluorimetry (DSF [30]) was performed on the above 50 prioritized hits in the absence and in the presence of the cofactor in its oxidized or reduced state (NAD^+^ or NADH, respectively) (Figures 3 and 4). The melting point, T_m_, of ligand-free 15-PGDH at pH 8.0 was 41.2±0.3°C (Figure 4A). The stability was increased by more than 4°C in the presence of NAD^+^ (T_m_ = 45.9±0.1°C) and by more than 10°C in the presence of NADH (T_m_ = 52.5±0.4°C). The substrate PGE_2_ failed to stabilize without cofactor, but stabilized by 2.4°C in the presence of NAD^+^ (Figure 4A). Interestingly, no stabilization was observed in the presence of NADH. Similarly to PGE_2_, none of the inhibitors stabilized the protein in the absence of cofactor (Figure 4A and supplementary information Table S1), suggesting that the cofactor may be necessary for the enzyme to assume a ligand-competent conformation. This observation is also in general concordance with the ordered bi-bi mechanism of the enzymatic reaction of short-chain dehydrogenases including 15-PGDH [31]. In the presence of cofactor, the 50 selected hits elicited a range of thermal stability enhancements up to 12.2°C in the presence of NAD^+^ and up to 13.5°C in the presence of NADH (expressed as a shift in the transition midpoint temperature, ΔT_m_) (Figure 3). A correlation between the ΔT_m_ and the inhibitory potency has been shown in a number of protein-ligand systems, particularly for kinases [32]–[34]. In the case of 15-PGDH, the correlation between inhibitory pIC_50_s and ΔT_m_ in the presence of cofactor appears to depend on chemotype: across the multiple clusters the correlation is generally weak (Figures 3A and 3B), while for individual chemotype clusters a meaningful correlation is observed. For the largest cluster 1, a moderate correlation is apparent in the presence of NAD^+^, while the correlation in the presence of NADH is more robust (R^2^ = 0.886): in general, compounds in this cluster show greater T_m_ shifts with NADH than with NAD^+^, as exemplified by the most potent analogue, compound **13**, [1-(3-methylphenyl)-1*H*-benzimidazol-5-yl)(piperidin-1-yl)methanone (a.k.a. ML148), (IC_50_ of 56 nM, ΔT_m_ = 7.3±0.2°C or 13.5±0.9°C with NAD^+^ or NADH, respectively). Compound **72** (a.k.a. ML149), belonging to a different cluster, showed a similar profile with a significantly larger ΔT_m_ in the presence of NADH. Conversely, members of the smaller cluster 5 showed the reverse profile: the most potent analogue **61** (a.k.a. ML147) gave an IC_50_ of 141 nM and T_m_ shifts of 12.2±0.1°C or 2.9±0.5°C with NAD^+^ or NADH, respectively.

### Verification of inhibitor activity after re-synthesis

The contrasting stabilization profiles alerted us to the possibility of differing mechanisms of inhibition, and we selected compounds **13**, **61** and **72** for further study. In order to definitively confirm identity and activity and to provide high quality material for in-depth kinetic studies, these three compounds were re-synthesized (see Materials and Methods). The effects of the re-synthesized compounds on the protein stability (Figure 4A) were found to be similar to the results obtained previously. To achieve an optimal signal window in the detailed kinetic measurements with the new compounds, a higher enzyme concentration was employed and the reactions were run at a lower percent substrate conversion than used in both the qHTS and the re-tests. As a result, higher apparent potencies were observed for all three compounds (Figure 4B): The IC_50_ for compound **13** was 7.9±0.8 nM, compared to 56 nM calculated according to the re-test results, and for compounds **61** and **72**, respectively, values of IC_50_ of 26.4±2.4 and 15.0±1.2 nM were determined (compared to 141 nM and 82 nM, respectively). All three compounds caused a maximum of 100% inhibition (Figure 4B).

### Catalytic constants in presence of inhibitors

In order to explore the mechanisms of action of **13**, **61** and **72**, we determined the dependence of the enzyme activity on the PGE_2_ substrate and NAD^+^ cofactor concentrations, first in the absence of inhibitor, and then at inhibitor concentrations close to their IC_50_, respectively (Table 1). The Michaelis constant, *K*_m_, for PGE_2_ was calculated from the data as 5.5±0.6 µM, comparing favorably with previously published values [21], [35], [36]. The maximal rate, *V*_max_, for the oxidation of PGE_2_ was 28.1 µmol/(min·mg), and the catalytic constant, *k*_cat_, was about 14 per second; the catalytic efficiency, *k*_cat_/*K*_m_, was 2.5·10^6^. Compound **61** had almost no effect on the *V*_max_ (Figure 4C), while it caused an increase in the *K*_m_, by 100% at 10 nM and 3-fold at 50 nM, suggesting a competitive mechanism of inhibition with respect to PGE_2_ [37]. Global fitting of the results to the four possible modes of inhibition (competitive, uncompetitive, noncompetitive and mixed-type inhibition) returned the highest probability for the mixed-type mode (Table 2). However, the fitting results are also consistent with a competitive mode for inhibition by compound **61**, particularly when the residual Chi^2^ value for this mode is compared to those of the other two compounds. Together with the observation from the Lineweaver-Burk analysis of the data (inset in Figure 4C), the most consistent mode of inhibition for compound **61** is competitive inhibition with respect to the substrate PGE_2_. On this basis, considering the concentration of the substrate in the experiment, the observed IC_50_ corresponds to an inhibition constant, *K*_i_, of approximately 5 nM [38].

**Table 1:** Investigation of the mechanism of action for selected inhibitors of human 15-PGDH.

	Conc. [nM]	Titration of PGE_2_				Titration of NAD^+^			
		*K*_m_ [µM]	*V*_max_[µmol/(min×mg)]	*k*_cat_[1/sec]	*k*_cat_/*K*_m_[×10^6^]	*K*_m_ [µM]	*V*_max_ [µmol/(min×mg)]	*k*_cat_[1/sec]	*k*_cat_/*K*_m_[×10^6^]
**13**	10.020.0	3.7±0.52.8±0.4	21.312.6	10.3±0.36.1±0.2	2.82.1	10.7±0.68.9±0.8	17.58.2	8.5±0.14.0±0.1	0.80.4
**72**	4.010.0	5.6±1.16.4±0.6	22.815.4	11.1±0.57.5±0.2	2.01.2	n.d.	n.d.	n.d.	n.d.
**61**	10.050.0	7.5±0.513.5±1.3	29.025.6	14.1±0.212.4±0.3	1.80.9	17.0±0.811.1±1.2	21.110.2	10.3±0.15.0±0.1	0.60.4
PGE_2_		5.5±0.6	28.1	13.6±0.3	2.5				
NAD^+^						15.8±0.9	21.2	10.3±0.1	0.7

**Table 2:** Global fitting of substrate titration data to different modes of inhibition (CM, competitive inhibition; UM, uncompetitive inhibition; NM, noncompetitive inhibition; MM, mixed-mode inhibition).

	PGE_2_			NAD^+^		
Compound	13	72	61	13	72	61
Competitive (CM)	0.8669	0.9299	0.9845	0.8764	n.d.	0.9507
Uncompetitive (UM)	0.9784	0.9680	0.9742	0.9664	n.d.	0.9837
Noncompetitive (NM)	0.9777	0.9734	0.9800	0.9655	n.d.	0.9834
Mixed-mode (MM)	0.9797	0.9736	0.9866	0.9675	n.d.	0.9841
Preferred*	MM	NM	MM	UM	N/A	UM
Sum-of-squares F-test	UM vs. MM:p = 0.0133	UM vs. NM:N/A$	CM vs. NM:N/A$	UM vs. NM: N/A$	N/A	UM vs. NM: N/A$
	NM vs. MM:p = 0.0017	NM vs. MM:p = 0.3515	CM vs. MM:p = 0.0001	UM vs. MM:p = 0.0654	N/A	UM vs. MM: p = 0.1058

Displayed are the fit residual Chi^2^ values, as well as probabilities of selected modes in comparison, from sum-of-squares F-testing.

*Analyses performed using PRISM 5.03 (GraphPad, Inc.). The threshold for rejection of the null-hypothesis (i.e., “The less complicated model is correct.”) was set to p = 0.05.

$ Both models with an equal number of degrees of freedom.

In contrast, both compounds **13** and **72** decreased the maximum rate of 15-PGDH in titrations of PGE_2_ (Table 1). Applied at a concentration of 10 nM, compound **13** decreased *V*_max_ by 25% (20 nM of the compound reduced *V*_max_ to less than half; Figure 4D), while compound **72** caused a loss of approximately 50% in maximum activity. Compound **13** additionally also reduced the apparent *K*_m_ by half at a concentration of 10 nM, suggesting that the affinity of the enzyme for the substrate was increased by the inhibitor, consistent with a mode of uncompetitive or mixed-type inhibition. Because both *V*_max_ and *K*_m_ were reduced, almost no change in the catalytic efficiency, *k*_cat_/*K*_m_, was observed with this compound. In contrast, Compound **72** showed no effect on the *K*_m_, and consequently also caused a reduction in the catalytic efficiency of the enzyme [37]. Comparative data fitting made a noncompetitive or mixed-type mode most likely for compound **72**, while the data for compound **13** are consistent with noncompetitive, uncompetitive or mixed-modes (Table 2).

The effects of compounds **13** and **61** on the rate constant and catalytic efficiency of NAD^+^ reduction were investigated: The *K*_m_ for NAD^+^ was lower than for PGE_2_ (15.8±0.9 µM), comparing favorably with published values [21]; the catalytic efficiency, *k*_cat_/*K*_m_, was determined as 0.7·10^6^ (Table 1). The decrease in the maximum rate of NAD^+^ reduction by compound **13** at 20 nM was similar to its effect on the conversion of the substrate PGE_2_: the *V*_max_ was decreased by approximately 60% and the *K*_m_ for NAD^+^ by ∼45%. Taken together, these data suggest that compound **13** acts noncompetitively or in a mixed-type mode with respect to the substrate while uncompetitively with respect to the cofactor. For compound **61**, on the other hand, a difference between the influence on PGE_2_ oxidation and on NAD^+^ reduction was observed: for the latter, this compound caused an effect similar to that of compound **13**, also suggesting an uncompetitive mechanism of action with respect to the cofactor (i.e., increasing the affinity of the enzyme for the cofactor). This interpretation was confirmed by the results of comparative fitting (Table 2) that showed uncompetitive inhibition with respect to the cofactor NAD^+^ as the most likely mode of inhibition for both compounds.

### Crystal structure of the 15-PGDH homodimer

**Figure 5:**
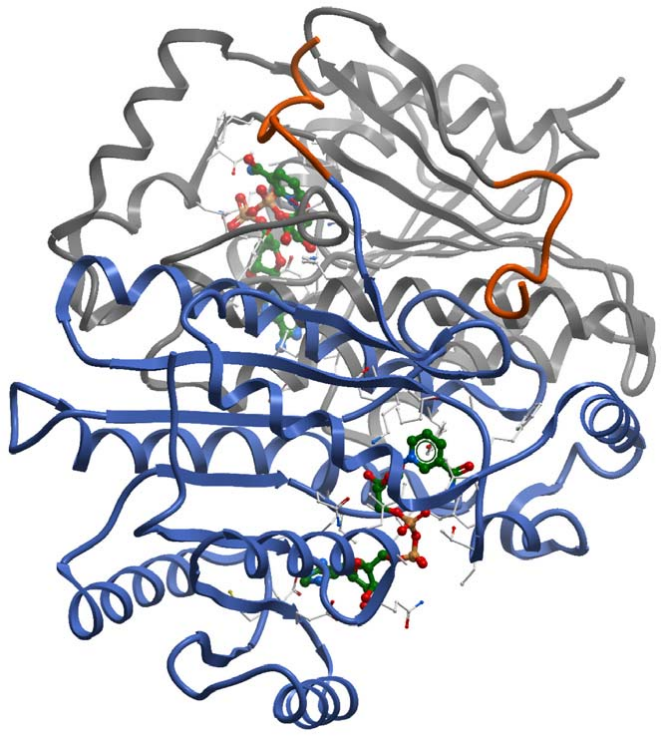
Structure of the homodimer of human 15-PGDH in complex with NAD^+^ (PDB: 2GDZ). Displayed are the two protomers with their backbones in grey and blue, respectively. The cofactor is depicted in a ball-and-stick representation and color-coded by atom type (green, carbon; red, oxygen; blue, nitrogen; orange, phosphate). The C-terminus of each protomer whose residues originate from the cloning procedure (see text) is highlighted in orange.

We were interested to explore whether the structure of 15-PGDH could provide insights into the observed mechanisms of the newly discovered inhibitors. After several unsuccessful crystallization attempts with constructs bearing different tags fused to the N- or C-terminus, respectively, it was possible to grow crystals in presence of NADH from a construct purified with a C-terminal His_6_ tag and to determine for the first time the structure of the homodimer of 15-PGDH in a complex with its cofactor at a resolution of 1.65 Å (Figure 5 and Supplemental Table S2). This resolution is not high enough to distinguish the reduced state of the cofactor from its oxidized form [39]. It is, however, unlikely that the structure of 15-PGDH contained NADH considering the time necessary to generate the crystals and the known short half-life of NADH in solution, despite the addition of the cofactor in its reduced state. The protein shows the α/β folding pattern that is highly conserved among the short-chain dehydrogenases/reductases (SDR), where a central β-sheet consisting of 8 strands is flanked on either side by two arrays of α-helices (Rossmann fold) [18], [40]. In the crystallized construct the last ten amino acids from the 15-PGDH sequence are missing, leaving Tyr256 as the last native amino acid. Instead, the cloning procedure resulted in the fusion of nine non-natural amino acids (Gly-Ser-Lys-Glu-Asn-Leu-Tyr-Phe-Gln) to the C-terminus remaining after cleavage of the tag. In the structure of the dimer the artificial C-terminus of one protomer is folded over the top of the opposing protomer (highlighted in orange in Figure 5), with a number of residues engaging in interactions with side chains of the other protomer, notably between Phe264 and Tyr217. One cofactor molecule per protomer is located in the center of the molecule (in ball-and-stick representation in Figure 5). The acidic residue Asp36 forms hydrogen bonds to the 2′- and 3′-hydroxyl groups of the adenine ribose moiety, while Asp64 forms a hydrogen bond to the amino group of the adenine moiety (supplementary information Figure S2). The nicotinamide moiety of NAD^+^ is positioned close to the catalytic tetrad (Asn107, Ser138, Tyr151 and Lys155), competent for the hydride transfer from the S-side of C4 to the substrate following the reaction mechanism conserved among all SDRs [31], [40]–[42].

### Docking of inhibitors to the 15-PGDH•NAD^+^ complex

**Figure 6:**
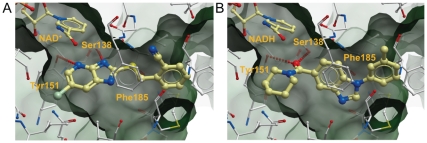
Binding of the inhibitors 61 (*A*) and 13 (*B*) in the active site of 15-PGDH as predicted by docking studies. The view shows a cut-through into the substrate pocket of 15-PGDH, with the volume of the pocket indicated by a green mesh. Key amino acid residues (see text) are labeled. Compounds and amino acid sidechains are in ball and stick representation, with the atoms color-coded: blue, nitrogen; red, oxygen; silver, bromide; white, carbon atoms in side chains; yellow, carbon atoms in compounds; green, carbon atoms in the cofactor. The figures were created using ICM (Molsoft, LLC).

All attempts to co-crystallize the inhibitors with 15-PGDH were unsuccessful. Therefore, we explored the inhibitor binding modes by computational docking of the compounds to the structure of the 15-PGDH•NAD^+^ complex. Docking experiments were carried out using the structure of a single protomer, to avoid the artifactual occupancy of the substrate pocket by the C-terminus of the opposing protomer (see above). This structure offers a large volume for binding of the inhibitors, notably a hydrophobic pocket close to the nicotinamide moiety of the NAD^+^, flanked by Ile195, Leu192, Ile211 and Ile215 (indicated by a green mesh in Figures 6A and 6B). An elongated tunnel leads to the exterior of the protein, formed by Phe185, Met213, Leu139, Met143 and Val145.

We identified plausible binding modes with well-defined side chain interactions which, interestingly, were similar for all three inhibitors, despite their structural differences (Figures 6A and 6B, supplementary information Figure S3). Binding of compound **61** appears to be driven by interaction of the imidazo-pyridine ring with the catalytic Ser138 and Tyr151 residues, while the bromo-substituent projects into the hydrophobic pocket. The cyanobenzyl group is accommodated in the substrate tunnel (Figure 6A). For compound **13**, the amide carbonyl also interacts with the catalytic residues Ser138 and Tyr151, while the piperidine ring fits snugly into the adjacent hydrophobic pocket. The remainder of the molecule is accommodated along the tunnel towards solvent, with the side chain of Phe185 providing a stacking opportunity for the central benzimidazole moiety of **13** (Figure 6B). The binding mode found for compound **72** is similar, with the triazole moiety mimicking the amide carbonyl of compound **13** in accepting hydrogen bonds from both Ser138 and Tyr151 (supplementary information Figure S3). The fused azepine ring occupies the proximal hydrophobic pocket, and the remaining biaryl moiety extends into the substrate tunnel, again with the possibility for stacking between the pyrrole ring of **72** and Phe185.

## Discussion

Selective, high-affinity inhibitors for human 15-hydroxyprostaglandin dehydrogenase (15-PGDH) are desirable as tools to facilitate the mapping of prostaglandin signaling pathways *in vitro* and *in vivo*. Using a quantitative high-throughput screen approach we have found several new chemotypes that inhibit 15-PGDH with high affinity. Detailed biophysical analyses have demonstrated the strong stabilizing effect of these molecules on the enzyme. Based on the results from our detailed kinetic analyses a number of inhibition modes appeared possible, but the data are consistent with compounds **13** and **72** not inhibiting competitively, while a competitive mechanism of inhibition appeared likely for compound **61**.

**Figure 7:**
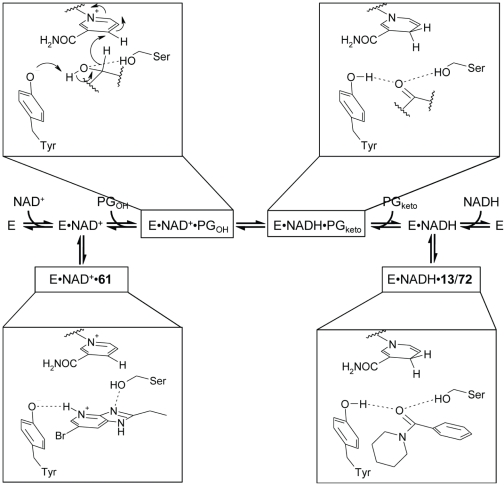
Proposed mechanism of prostaglandin dehydrogenation (after [**31**]) and inhibitory mechanisms of action for compounds 61 and 13. Compound **61** mimics the substrate (left panels), consistent with experimental data showing favored binding to the NAD^+^-bound form of 15-PGDH, and its competitive mechanism of inhibition with respect to PGE_2_. Compound **13** mimics the product of oxidation (right panels), consistent with its favored binding to the NADH-bound form of 15-PGDH, and its uncompetitive mechanism of inhibition with respect to PGE_2_.

In order to gain better understanding of the distinct mechanisms of action of the inhibitors, it is helpful to examine the catalytic mechanism of 15-PGDH. The SDR enzymes have been shown to follow a common sequential ordered bi-bi-reaction mechanism, involving sequential cofactor binding, substrate binding, catalysis, product release, and finally release of co-product [31], [40], as shown in the reaction coordinate (Figure 7). In analogy with other SDRs, binding of the NAD^+^ cofactor is expected to alter the local electrostatic environment of the catalytic Tyr151 residue, favoring the deprotonated state. After substrate binding, the reaction proceeds by deprotonation of the substrate 15-OH group by Tyr151, facilitating hydride transfer from the substrate to NAD^+^, and forming the product complex consisting of protonated Tyr151, NADH, and ketone.

One of the inhibitor chemotypes identified from the high throughput screen is a series of imidazopyridines, represented by the most potent analogue **61**, with an IC_50_ of 25 nM and an estimated *K*_i_ of 5 nM. The biophysical data show a strong preference for **61** in stabilizing 15-PGDH complexed with NAD^+^ as compared with NADH.

The results of the docking experiments provide support for a competitive mechanism of inhibition by compound **61**. The docking directs the imidazopyridine group into hydrogen bonding interactions with the catalytic residues Tyr151 and Ser138. In the co-complex with NAD^+^, as discussed above, the deprotonated form of Tyr151 is favored: the resultant oxyanion is able to interact with the protonated form of the pyridine ring, while the hydroxy group of Ser138 is able to donate a hydrogen bond to the adjacent imidazole nitrogen. The much weaker stabilization observed with NADH implies that the protonated form of Tyr151 interacts considerably more weakly with the pyridyl nitrogen.

In addition to providing a convincing basis for interaction of the heterocyclic motif with the enzyme's catalytic machinery, this binding orientation also favorably directs the bromo-substituent into the nearby hydrophobic pocket, and places the S-linked cyanobenzyl group into the substrate tunnel leading towards the exterior of the protein, which contains side chains available for stacking interactions (Phe185, Tyr217). Taken together, the data argue that the position for binding of compound **61** along the reaction coordinate of 15-PGDH is at step 2, i.e., occurs to the complex of 15-PGDH•NAD^+^ (left panel in Figure 7). This interpretation is consistent with the observed uncompetitive mechanism of action with respect to NAD^+^, since the higher affinity of the inhibitor compared to the substrate is expected to increase the affinity for NAD^+^.

The majority of the remaining inhibitor hits fall into three structural clusters (clusters 1, 2 and 4; supplementary information Table S1), all containing an amide moiety as the common feature (Figure 2). Structure-activity relationships indicate that activity is favored when the nitrogen of the amide forms part of a saturated heterocycle, most commonly piperidine. In all of the active amides identified, the carbonyl is linked to a ring, which is aromatic in the most potent analogues. The highest potency is achieved when this ring is linked to a further aryl ring, with a variety of linkers being tolerated. In compound **13**, this linker is incorporated into the central fused benzimidazole ring system.

The results of the docking experiments are consistent with the observed structure-activity relationships and provide help with the decision about which of the mechanisms of inhibition that are possible according to the kinetic analyses would be most likely for the inhibitors. In the model of compound **13** docked into 15-PGDH, the oxygen of the amide carbonyl presents two lone pairs that can accept hydrogen bonds from both the catalytic Ser138 and Tyr151 residues, mimicking the ketone product of oxidation of the PGE_2_ substrate. Tyr151 is only able to donate this hydrogen bond when protonated, which is favored by binding of NADH, while binding of NAD^+^ favors the deprotonated state. Hence, compound **13** acts as a product analogue in the sequential ordered bi-bi catalysis mechanism, and a noncompetitive mode of inhibition with respect to PGE_2_ and an uncompetitive mode with respect to NAD^+^ would be expected [43]. Our kinetic analysis is consistent with this mode, identifying uncompetitive inhibition with respect to the cofactor, while equal probability was found for noncompetitive, uncompetitive and mixed-type inhibition with respect to the substrate (Table 2). Uncompetitive inhibition by the compounds of the series with respect to NAD^+^ is, furthermore, consistent with the observed enhanced stabilization of 15-PGDH when the latter is bound to NADH as compared with NAD^+^; this stabilization of product-bound 15-PGDH ultimately results in a suppression of enzymatic turnover.

The preference for small alicyclic amides is also explained by the docking model: the piperidine moiety of compound **13** is relatively tightly accommodated in the hydrophobic pocket adjacent to the catalytic residues (Figure 6B). Consistent with this binding mode, inhibitory potency is weaker for secondary or tertiary straight-chain amides, and further diminished or abolished with bicyclic amides that can clearly not be accommodated in this pocket (see Figure 2). As described above, the structure-activity relationships indicate that activity is retained with a variety of groups attached to the amide carbonyl carbon atom, although there is a preference for aromatic groups, and in general these groups are substituted in such a way as to be able to adopt an extended conformation. Such a conformation is consistent with the docking mode, placing the extended group into the substrate tunnel leading towards the exterior of the protein. The availability of side chain groups capable of stacking interactions (Phe185, Tyr217) adds further support to this proposed binding mode (Figure 6).

Compound **72** shares aspects of its mechanism with compound **13**, notably a strong preference for NADH in co-stabilization of 15-PGDH (Figures 3 and 4A) and a cofactor-uncompetitive inhibition mechanism. With respect to the substrate PGE_2_ the kinetic analysis favored the noncompetitive mode, consistent with the expectation for a product-analogous binding mode (see above). Although compound **72** does not contain an amide group, the fused 1,2,4-triazole is capable of acting as a bioisostere, with a lone pair of electrons on each of the adjacent nitrogen atoms mimicking the two lone pairs of the carbonyl oxygen in **13** (Supplementary Information Figure S3). Thus, in the docking experiments, the triazole moiety accepts two hydrogen bonds from Ser138 and the protonated form of Tyr151; the remaining hydrophobic and stacking interactions are remarkably similar to those modeled for compound **13**. Therefore, both compounds **13** and **72** bind at step 4 along the 15-PGDH reaction coordinate (Figure 7), mimicking the product, favoring co-complex formation with NADH and behaving in an uncompetitive manner with respect to the PGE_2_ substrate.

The compound library used for screening of 15-PGDH in this study has been, so far, tested in screens against over 320 targets (http://pubchem.ncbi.nlm.nih.gov/). This list includes two other proteins that are involved in prostaglandin signaling, the EP2 prostaglandin-E_2_ receptor and the M1-muscarinic receptor. The list also includes two SDR enzymes, hydroxyacyl dehydrogenase (HADH2, also known as type-10 hydroxysteroid dehydrogenase, HSD17β10; PubChem Assay ID 886) and type-4 hydroxysteroid dehydrogenase (HSD17β4; PubChem Assay ID 893), and a medium-chain dehydrogenase/reductase (MDR), aldehyde dehydrogenase 1 (ALDH1A1; PubChem Assay ID 1030), each of which was tested using a similar experimental protocol to that used for 15-PGDH. As the results in Table 3 show, all lead inhibitors identified in this study were largely inactive against ALDH1A1: compounds **61** and **72** showed a flat response, while compound **13** displayed only a shallow curve with an approximate IC_50_ of 36 µM. Against the two hydroxysteroid dehydrogenases (HSD17β10 and HSD17β4), both compounds **13** and **72** were inactive up to the top concentration of 57.5 µM.

**Table 3:** Selectivity profiling of key inhibitors against related dehydrogenase/reductase enzymes.

Compound	CID	IC_50_/nM			
		15-PGDH	ALDH1A1	HADH2	HSD17β4
13	4249877	56	36000*	>57500	>57500
72	3717642	89	>57500	>57500	>57500
61	4251360	141	>57500	n.d.	n.d.

*Maximal response 70% inhibition.

In summary, the new inhibitor chemotypes identified in this study provide a set of tools, with complementary mechanisms of inhibition, for functional studies on the role of 15-PGDH in prostaglandin signaling pathways. The lead compounds that have been characterized in detail exhibit nanomolar affinities. The existence of disease scenarios involving over-activation of 15-PGDH and in which selective inhibition of the enzyme would be beneficial, is currently unclear. Work published by Tai and co-workers suggested that certain prostate cancers might involve higher-than-normal activity of 15-PGDH [44], and for such cases application of inhibitors based on the chemotypes identified in this study may prove useful. The potential utility of the probes is reinforced by the low cross-reactivity of these compounds in screens against multiple targets, among them receptors with a role in prostaglandin signaling and dehydrogenase enzymes utilizing the same cofactors, suggesting selectivity of the inhibitors for 15-PGDH. The availability of multiple inhibitors with diverse chemical structures for cellular studies enhances confidence in any response observed being due to on-target effects; the likelihood for such diverse compounds having an identical off-target activity profile is very small. While it is difficult to anticipate differences in the cellular response between inhibitors showing contrasting modes of action, one might expect the noncompetitive compounds to demonstrate inhibition independently of the substrate concentration. This may help to avoid problems similar to those encountered, for example, with ATP-competitive protein kinase inhibitors, where high inhibitor concentrations are required in cells to compete against the endogenous co-factor. Finally, in this work we present the novel crystal structure of human 15-PGDH, which will serve as a basis for further studies on the function, mechanism and inhibitor design for this enzyme.

## Materials and Methods

### Cloning, Expression and Purification

The protein was expressed in *E. coli* BL21(DE3)-R3 cells, from a synthetic DNA sequence comprising residues 3–256 of the human HPGD gene (GenBank identifier: 1203982). Cloning into the p15 vector added a TEV-cleavable (*) C-terminal His_6_ tag (gskenlyfq*ghhhhhh) and four extra amino acids, MAHM, at the N-terminus. The protein was purified using immobilized Ni-affinity chromatography followed by proteolytic cleavage of the His_6_ tag and size exclusion chromatography. As judged by SDS-PAGE, the sample was >95% pure; the correct molecular weight was confirmed using electrospray mass spectrometry. The protein was frozen in aliquots in liquid nitrogen and stored at −80°C until use.

### Quantitative high-throughput screening (qHTS)

The 160,182-member library consisted of compounds from the National Institutes of Health Molecular Libraries Small Molecule repository (NIH MLSMR), prepared as 10 mM stock solutions in 384-well plates and delivered by Galapagos Biofocus DPI (South San Francisco, CA, http://mlsmr.glpg.com), from the NCGC internal exploratory collection comprising several commercially available libraries of known bioactives, as well as from libraries provided by commercial and academic collaborators. Details on the library composition and its formatting for qHTS have been published elsewhere [27], [29], [45].

The enzymatic reaction was performed in 50 mM Tris-HCl pH 8.0, containing 0.01% Tween 20. Final reactions comprised 20 nM 15-PGDH, 1 mM NAD^+^ and 30 µM PGE_2_. The high-throughput screen was performed on a fully integrated robotic system (Kalypsys Inc, San Diego, CA) as described elsewhere [45]. Library compounds, arrayed as seven-point inter-plate concentration series [29], were screened from the lowest (3.5 nM) to the highest (57.5 µM) concentration, with plates containing DMSO only, serving to note any systematic drifts in the signal, interspersed evenly throughout the screen (approximately every 50 plates). Briefly, 3 µL of reagents were dispensed into 1536-well Greiner black solid-bottom assay plates using integrated nanoliter solenoid-technology dispensers [45]. Compounds and controls (23 nL) were transferred via Kalypsys pintool equipped with 1,536-pin array (10-nL slotted pins; V&P Scientific, Palo Alto, CA). After a 15-minute incubation (to allow for interaction between compound and enzyme), a 1-µL substrate addition step initiated the reaction. Kinetic data were collected on a ViewLux High-throughput CCD imager (Perkin-Elmer) equipped with standard UV fluorescence optics (exc. 340 nm, em. 450 nm).

Starting fluorescence intensity and the change in fluorescence over the two-minute initial rate period were recorded for every well. Screening data were normalized against no-enzyme (64 wells, columns 3 and 4) and no-compound (32 wells located in column 1) controls, and concentration-response relationships were derived as described previously [46]. A four-parameter Hill equation [47] was fitted to the concentration-response data using publicly available algorithms (http://www.ncgc.nih.gov/pub/openhts/curvefit/). Similarity clustering of active compounds and SAR (structure-activity relationship) derivation were performed as described previously [46].

### Kinetic analysis and inhibitor mechanism of action studies

Kinetic studies were performed in 100 mM Tris buffer pH 8.0, containing 0.01% Tween-20 and 10 nM 15-PGDH enzyme. NAD^+^ and PGE_2_ were applied from stock concentrations of 100 or 2 mM, respectively, in 500 mM Tris/HCl, pH 8.0. All inhibitor compounds were dissolved in 10% (v/v) DMSO at varying concentrations from which they were applied at 1∶5 ratio (resulting in a final concentration of 2% (v/v) DMSO in all assays). For each experiment an aliquot of purified enzyme was thawed freshly and pre-diluted to 10 µM in buffer I (5 mM Tris/HCl, pH 8.0, 150 mM NaCl, 0.01% Tween-20). All experiments were performed in white 384-well PCR microplates (Bio-Rad Laboratories, Inc.) in final assay volumes of 20 µL. Twelve-point compound titrations were set up from serially diluted stock solutions in 10% (v/v) DMSO. Before starting the reactions by addition of 30 µM PGE_2_ substrate and 200 µM NAD^+^ in buffer I (from injectors built into the spectrometer, PolarStar Omega, BMG Labtech GmbH), the compounds were incubated in the wells of the plate with enzyme for ten minutes at the experimental temperature of 25°C. Experiments comprising substrate titrations, from serially diluted stock solutions in buffer I, were started by injection of buffer I containing enzyme and NAD^+^, while cofactor titrations were set up in assay buffer containing 30 µM PGE_2_ substrate and started by injection of enzyme in buffer I. The reduction of NAD^+^ was followed by monitoring fluorescence emission at 460 nm, excited at 355 nm (10 nm bandwidth filters, PolarStar Omega, BMG Labtech GmbH), at a reading frequency of 22 s/point or 12 s/point for compound- or substrate/cofactor titrations, respectively. The initial rates recorded in compound titrations were plotted against -log[compound], and non-linear regression (Levenberg-Marquardt) fitting of the dose-response curve to the Hill equation [47] was performed to derive the IC_50_, using PRISM 5.0 (GraphPad Software, Inc.). For substrate/cofactor titrations, curves with initial rates plotted against the concentration of substrate or cofactor were fitted to the Michaelis-Menten equation, to derive maximal activities, *V*_max_, and Michaelis constants, *K*_m_. Fitting qualities were judged from the goodness of fit (R^2^>0.98).

### Differential Scanning Fluorimetry (DSF)

Studies to assess the thermal stability of human 15-PGDH in presence of compounds were performed as described previously [30], with variations. Briefly, the protein was diluted to a final assay concentration of 1 µM in 100 mM Tris buffer pH 8.0, containing 0.01% Tween-20 and 1∶1000 SYPRO orange dye (Invitrogen). The final assay volume was 10 µL, with or without 200 µM of either NAD^+^ or NADH. Compounds were added at a ratio of 1∶5 from appropriate stock solutions in 10% (v/v) DMSO to comprise either 50 or 250 µM final concentrations. Heat denaturation curves were recorded using a RT-PCR instrument (Mx3005p, Agilent) applying a temperature gradient of 2°C/min. Analysis of the data was performed using DSF ANALYSIS 2.5 (ftp://ftp.sgc.ox.ac.uk/pub/biophysics) and PRISM 5.0 (GraphPad Software, Inc.).

### Chemical Synthesis of compounds

Compounds were synthesized following established procedures, from starting materials and reagents purchased from Aldrich Chemical Co. (supplementary information Text S1). The identity of the compounds was confirmed by analytical thin layer chromatography (TLC), nuclear magnetic resonance (NMR) and high-resolution mass spectrometry.

### X-ray crystallography

For crystallization of human 15-PGDH, purified protein was concentrated by ultrafiltration to 17 mg/mL (Vivaspin, cut-off: 30 kDa). Prior to crystallization NADH (Sigma) was added to a concentration of 5 mM to the concentrated protein in a volume of 100 nL. The solution was then mixed with 100 nL of crystallization solution containing 30% (w/v) PEG-1000 and crystals were obtained at 4°C using the sitting drop vapour diffusion technique. Crystals were transferred to a cryo-protectant consisting of a fifth of 100% (v/v) glycerol and four fifths of well solution, and flash-frozen in liquid nitrogen. A dataset extending to a resolution of 1.65 Å was collected on an R-AXIS HTC imaging plate area detector mounted on an F-RE SuperBright rotating anode generator (Rigaku MSC) operating at 45 kV and 45 mA (supplementary information Table S2). Data were indexed, integrated and scaled using MOSFLM v6.2.5 [48] and SCALA v5.0 (implemented in the CCP4 suite [49]). Initial phases were calculated by molecular replacement implemented by PHASER v1.3.1 [50] using an ensemble of three SDR structures (PDB codes 1WMB [51], 1IY8 [52] and 1ZK3 [53]) as search models over 50 cycles of automated model building using ARP/wARP [54]. Water molecules were automatically picked by ARP/wARP and later checked manually for appropriate density and hydrogen-bonding pattern. The final rounds of manual model building and refinement were carried out in COOT [55] and REFMAC v5.02.0005 [56], respectively (supplementary information Table S2). The coordinates of the final model and structure factors were deposited with the PDB accession ID 2GDZ. The structural information in this article can also be viewed as an enhanced version in which the text of the article is integrated with interactive 3D representations and animated transitions, available on the PLoS website (Datapack S1). A web plugin is required to access this enhanced functionality. Instructions for the installation and use of the web plugin are available in supplementary information Text S2.

### Molecular Docking

Docking was performed using GLIDE (Schrodinger, LLC) [57] and the structure of 15-PGDH was prepared using the software's default minimization protocols. Docking grids were generated using a box around Ser138 in the catalytic site. For docking to the NAD^+^ complex, Tyr151 was kept de-protonated; for docking to the NADH complex NAD^+^ was changed to NADH with a OPLS-2005 force field [58] and a hydrogen constraint to Tyr151 was used. Ligands (substrate and inhibitors) were prepared with the LIGPREP and IONIZER tools in GLIDE, at pH 7.0+/−2.0, to determine the ionization states of the ligands. All inhibitors were docked using GLIDE-SP and the top 5 scored poses were kept. The binding poses were visually inspected and hydrogen bond patterns were analyzed in order to determine the best pose.

## Supporting Information

Table S1 Structural clustering of 87 compounds re-tested after qHTS, with their potency in 24-point titrations compared to their effects on 15-PGDH thermal stability.
(0.15 MB PDF)

Table S2 Data collection and structural refinement statistics for human 15-PGDH.
(0.11 MB PDF)

Figure S1 Miniaturized screening assay for 15-PGDH. A. Activity heatmaps showing the assay miniaturization to a 4 µl volume in 1536-well format (increasing concentrations to the right). B. Plot of the Z' factor associated with each plate of the pilot concentration-response screen of the LOPAC1280 library performed to validate hit reproducibility. C. Molecular structure of the control inhibitor GW5074 that was added as a 16-point dilution series in duplicate between 57.5 µM and 1.75 nM into the second column of every assay plate (panel D). The average IC50 for the compound was 10.4 µM and the associated minimum significant ratio was approx. 1.5. E. Example of a fluorescent inhibitor (applied at between 3.5 nM and 57.5 µM) on the time course of NAD+-reduction upon injection of PGE2.
(0.28 MB PDF)

Figure S2 Binding of the cofactor NAD+ in the active site of human 15-PGDH. The acidic residue Asp36 forms hydrogen bonds to the 2′- and 3′-hydroxyl groups of the adenine ribose moiety, while Asp64 forms a hydrogen bond to the amino group of the adenine moiety. The nicotinamide moiety of NAD+ is positioned close to the catalytic tetrad (Asn107, Ser138, Tyr151 and Lys155).
(0.08 MB PDF)

Figure S3 Binding of inhibitor 72 in the active site of 15-PGDH as predicted by docking studies. The view shows a cut-through into the substrate pocket of 15 PGDH, with the volume of the pocket indicated by a green mesh. Key amino acid residues are labelled. The figures were created using ICM (Molsoft, LLC).
(0.07 MB PDF)

Text S1 Methods used in the chemical syntheses of the three lead compounds.
(0.17 MB PDF)

Text S2 Instructions for installation and use of the required web plugin (to access the online enhanced version of this article).
(0.46 MB PDF)

Datapack S1 Standalone iSee datapack - contains the enhanced version of this article for use offline. This file can be opened using free software available for download at http://www.molsoft.com/icm_browser.html.
(ICB)
