# Supplementary material for: High-Affinity Inhibitors of Human NAD+-Dependent 15-Hydroxyprostaglandin Dehydrogenase: Mechanisms of Inhibition and Structure-Activity Relationships
Source: PLoS One. 2010 Nov 2;5(11):e13719. doi: 10.1371/journal.pone.0013719 (PMC2970562; doi:10.1371/journal.pone.0013719)
Supplement: Table S1 — Structural clustering of 87 compounds re-tested after qHTS, with their potency in 24-point titrations compared to their effects on 15-PGDH thermal stability. (0.15 MB PDF) [file pone.0013719.s001.pdf]

High-affinity Inhibitors of Human NAD$^+$-dependent 15-Hydroxyprostaglandin Dehydrogenase: Mechanisms of Inhibition and Structure-activity Relationships

Frank H. Niesen, Lena Schultz, Ajit Jadhav, Chitra Bhatia, Kunde Guo, David J Maloney, Ewa S. Pilka, Minghua Wang, Udo Oppermann, Tom D. Heightman and Anton Simeonov

SUPPLEMENTARY INFORMATION TABLE S1

Structural clustering of 87 compounds re-tested after qHTS, with their potency in 24-point titrations compared to effects on stability of 15-PGDH. The structures were drawn using CHEMSKETCH v12.01 (Advanced Chemistry Development, Inc.).

No.	CID	Structure	Cluster	qHTS IC_{50} [nM]	ΔT_m [°C]		
					apo	NAD^+	NADH
1	858498	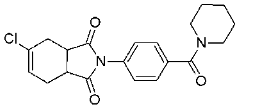	1	251	-0.1 ± 0.5	3.0 ± 0.3	6.1 ± 0.9
2	14733700	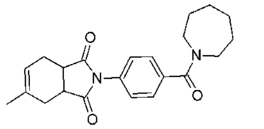	1	794	-0.6 ± 0.3	1.7 ± 0.2	6.0 ± 0.0
3	22412640	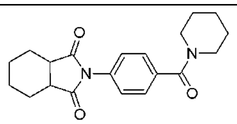	1	562	-0.6 ± 1.2	2.1 ± 0.7	6.7 ± 0.3
4	14733914	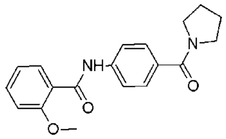	1	1000	0.3 ± 0.2	2.2 ± 0.0	4.7 ± 0.5
5	17508772	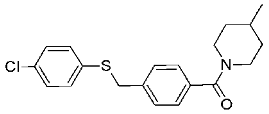	1	631	-0.8 ± 0.4	2.3 ± 0.2	7.1 ± 0.5
6	22404010	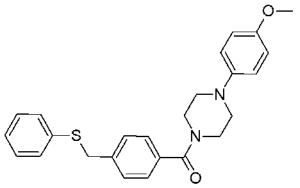	1	Inactive	n.d.	n.d.	n.d.
7	17386203	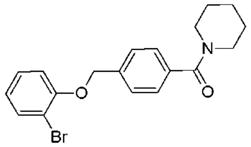	1	89	-0.1 ± 0.2	7.1 ± 0.4	10.6 ± 1.8
8	7974186	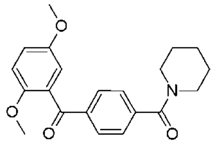	1	200	-1.4 ± 0.3	3.0 ± 0.1	6.6 ± 0.3
9	22404474	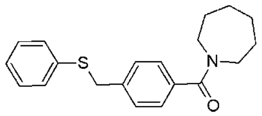	1	224	-1.1 ± 0.4	2.2 ± 0.1	7.8 ± 0.1

No.	CID	Structure	Cluster	qHTS IC_{50} [nM]	ΔT_m [°C]		
10	4247176	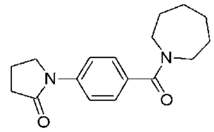	1	282	n.d.	n.d.	n.d.
11	17388363	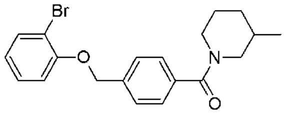	1	126	-0.4 ± 1.3	3.8 ± 2.1	10.6 ± 0.8
12	17415013	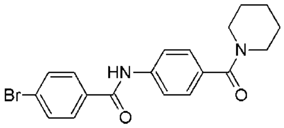	1	355	-1.6 ± 0.4	2.2 ± 0.4	7.1 ± 0.2
13	4249877	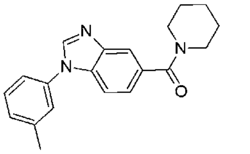	1	56	-1.7 ± 0.1	7.3 ± 0.4	13.5 ± 0.9
14	4242835	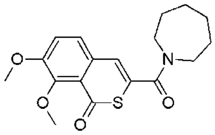	1	251	-1.0 ± 0.6	3.2 ± 0.1	7.8 ± 0.8
15	11113881	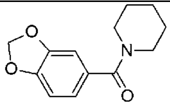	1	63	n.d.	n.d.	n.d.
16	7975310	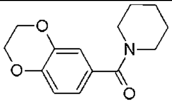	1	224	-1.8 ± 0.3	3.5 ± 0.0	7.8 ± 0.7
17	859816	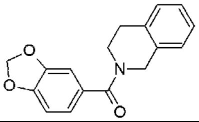	1	708	0.1 ± 0.1	3.3 ± 0.7	4.4 ± 1.2
18	14735329	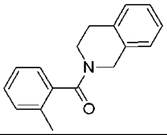	1	2512	-1.1 ± 0.4	1.5 ± 0.4	2.2 ± 0.2
19	4242245	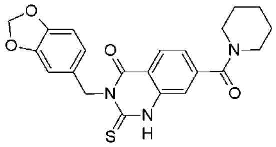	1	708	n.d.	n.d.	n.d.
20	17408510	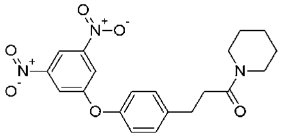	1	224	0.3 ± 0.2	3.1 ± 0.0	7.9 ± 0.9
21	17508174	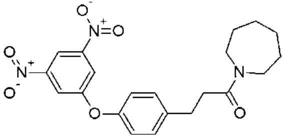	1	398	n.d.	n.d.	n.d.

No.	CID	Structure	Cluster	qHTS IC_{50} [nM]	ΔT_m [°C]		
22	17508775	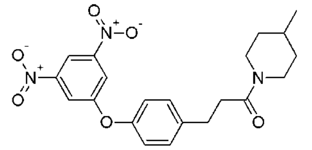	1	891	n.d.	n.d.	n.d.
23	17510669	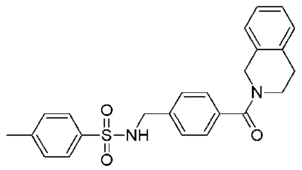	1	2239	-0.4 ± 0.6	1.2 ± 0.0	1.6 ± 0.2
24	17413567	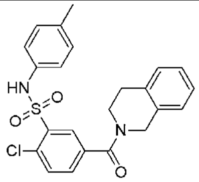	1	Inactive	n.d.	n.d.	n.d.
25	4257205	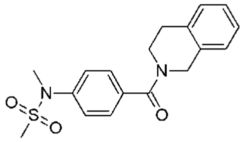	1	12589	n.d.	n.d.	n.d.
26	14740436	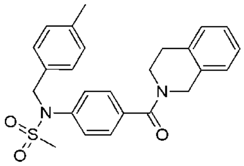	1	11220	n.d.	n.d.	n.d.
27	4258278	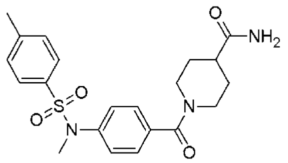	1	15849	n.d.	n.d.	n.d.
28	4256913	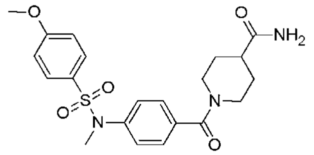	1	inactive	n.d.	n.d.	n.d.
29	17415524	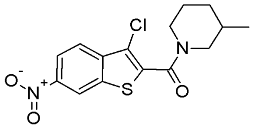	1	501	-0.1 ± 0.1	2.7 ± 1.4	8.4 ± 0.0
30	17434118	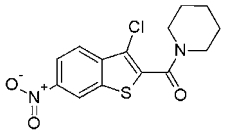	1	562	0.2 ± 0.0	2.4 ± 0.7	6.4 ± 0.0
31	14721743	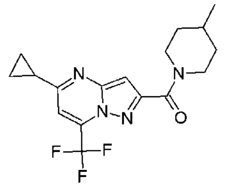	1	3981	-0.1 ± 0.1	1.1 ± 0.3	4.8 ± 0.1

No.	CID	Structure	Cluster	qHTS IC_{50} [nM]	ΔT_m [°C]		
32	14739308	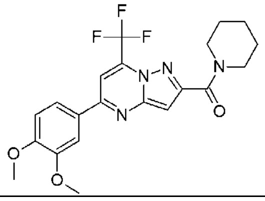	1	282	-1.4 ± 0.4	3.9 ± 0.6	9.6 ± 0.4
33	4264037	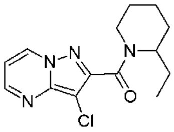	1	inactive	n.d.	n.d.	n.d.
34	22400460	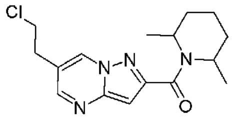	1	794	0.1 ± 0.5	2.0 ± 0.6	6.4 ± 0.0
35	4258044	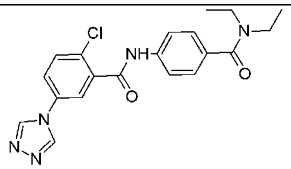	1	7080	-0.1 ± 0.4	1.2 ± 0.2	2.2 ± 0.1
36	14741020	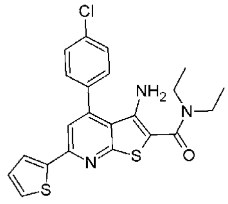	1	10000	-1.5 ± 0.1	0.9 ± 0.5	2.9 ± 0.4
37	17387444	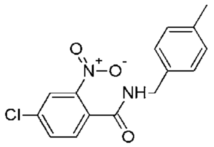	2	1995	-2.0 ± 0.2	1.1 ± 0.3	5.0 ± 0.1
38	17507886	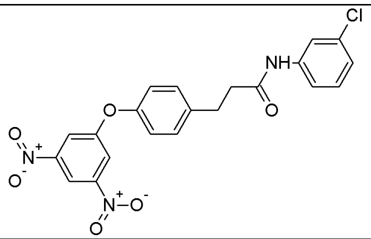	2	Inactive	n.d.	n.d.	n.d.
39	17386899	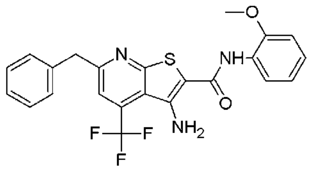	2	inactive	n.d.	n.d.	n.d.
40	14730699	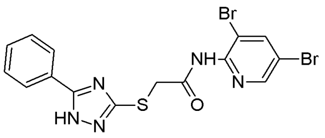	2	1413	n.d.	n.d.	n.d.

No.	CID	Structure	Cluster	qHTS IC_{50} [nM]	ΔT_m [°C]		
41	17509331	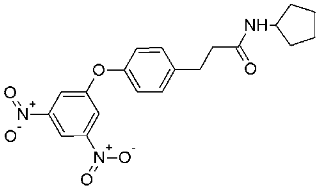	2	5012	n.d.	n.d.	n.d.
42	847763	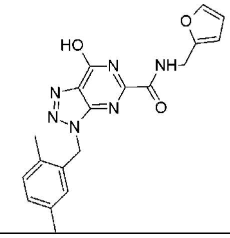	2	316	-0.5 ± 0.3	7.5 ± 0.1	4.8 ± 0.3
43	846052	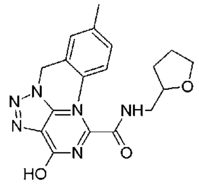	2	398	n.d.	n.d.	n.d.
44	14722954	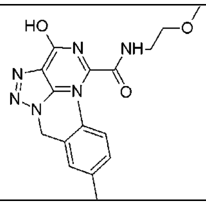	2	708	0.0 ± 1.2	8.9 ± 0.6	6.6 ± 0.4
45	14734193	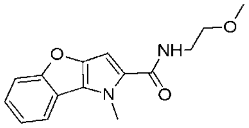	2	1585	-1.9 ± 0.8	2.7 ± 0.1	2.9 ± 0.1
46	865105	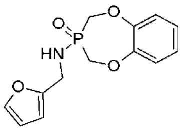	3	1000	n.d.	n.d.	n.d.
47	865424	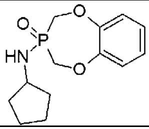	3	159	n.d.	n.d.	n.d.
48	865706	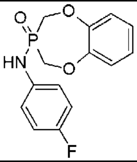	3	1000	-0.6 ± 1.2	0.5 ± 0.7	3.3 ± 0.1
49	865632	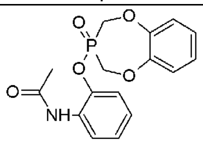	3	Inactive	n.d.	n.d.	n.d.
50	22411753	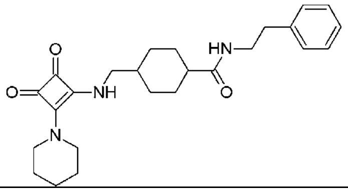	4	6310	-1.9 ± 1.5	1.9 ± 0.0	1.7 ± 0.0

No.	CID	Structure	Cluster	qHTS IC_{50} [nM]	ΔT_m [°C]		
51	22411616	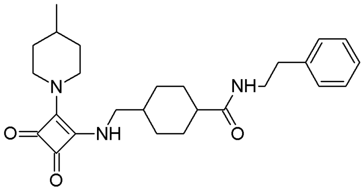	4	6310	n.d.	n.d.	n.d.
52	22411757	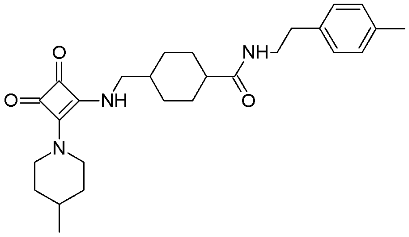	4	14125	n.d.	n.d.	n.d.
53	22411754	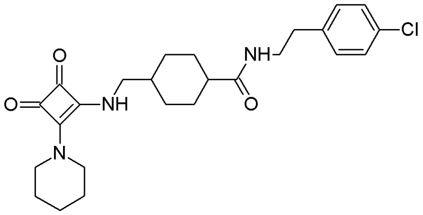	4	17783	n.d.	n.d.	n.d.
54	4247891	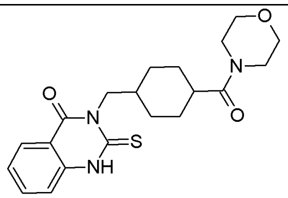	4	2512	-1.5 ± 0.3	6.0 ± 0.4	3.9 ± 0.2
55	4259277	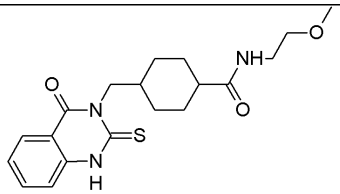	4	1122	0.1 ± 0.5	3.7 ± 0.7	4.7 ± 0.2
56	22416646	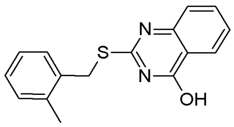	5	1000	0.0 ± 1.3	6.1 ± 1.7	0.4 ± 0.3
57	7973556	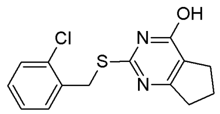	5	2239	-0.3 ± 0.2	9.2 ± 0.3	1.6 ± 0.1
58	14743576	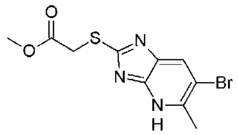	5	15849	n.d.	n.d.	n.d.
59	17513010	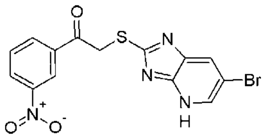	5	2239	-6.1 ± 2.4	6.9 ± 0.2	1.7 ± 0.3

No.	CID	Structure	Cluster	qHTS IC_{50} [nM]	ΔT_m [°C]		
60	14746455	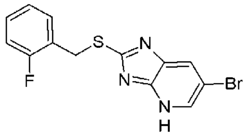	5	631	-0.2 ± 0.2	7.6 ± 1.9	1.5 ± 0.2
61	4251360	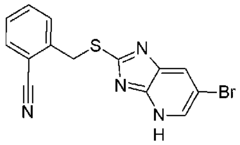	5	141	-1.2 ± 1.1	12.2 ± 0.1	2.9 ± 0.5
62	14740692	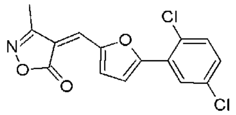	6	447	N/A	N/A	N/A
63	17504927	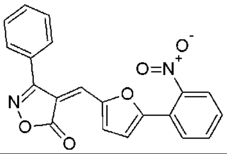	6	447	N/A	N/A	N/A
64	14737268	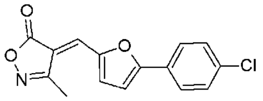	6	1122	N/A	N/A	N/A
65	17504829	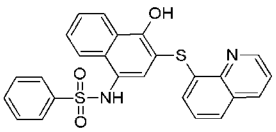	7	631	N/A	N/A	-0.2 ± 0.4
66	16952410	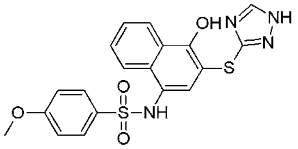	7	355	N/A	N/A	-0.7 ± 0.6
67	17433753	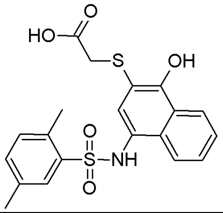	7	398	n.d.	n.d.	n.d.
68	17387000	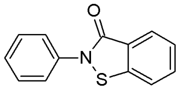	8	447	n.d.	n.d.	n.d.
69	17433179	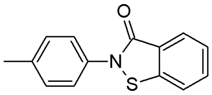	8	562	n.d.	n.d.	n.d.
70	3717771	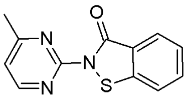	8	3162	n.d.	n.d.	n.d.

No.	CID	Structure	Cluster	qHTS IC_{50} [nM]	ΔT_m [°C]		
71	14746708	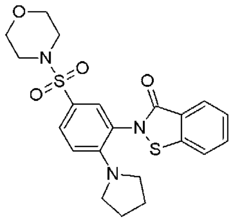	8	2512	-1.8 ± 1.4	2.4 ± 0.6	-0.4 ± 0.3
72	3717642	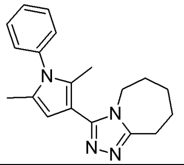	9	89	0.1 ± 0.1	5.0 ± 0.1	10.5 ± 0.6
73	3711888	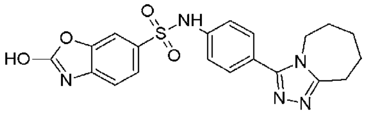	9	126	n.d.	n.d.	n.d.
74	22411855	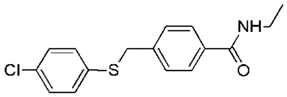	10	3981	n.d.	n.d.	n.d.
75	4238732	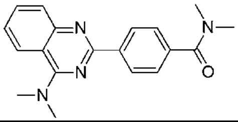	10	2512	n.d.	n.d.	n.d.
76	4239167	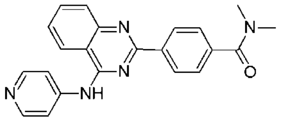	10	112	n.d.	n.d.	n.d.
77	4239829	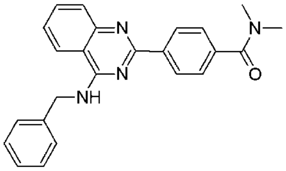	10	14125	n.d.	n.d.	n.d.
78	17408678	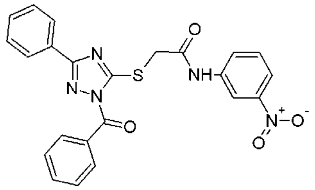	11	2512	-7.4 ± 2.5	4.0 ± 0.5	-7.6 ± 1.5
79	863921	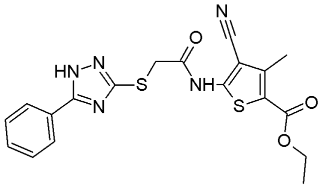	11	1122	-4.1 ± 2.2	N/A	N/A
80	14739621	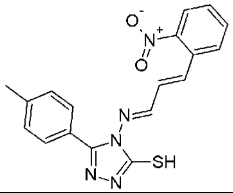	11	501	N/A	4.5 ± 0.2	0.2 ± 0.2

81	17513726	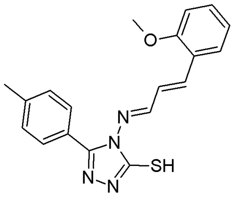	11	1585	n.d.	n.d.	n.d.
82	22403446	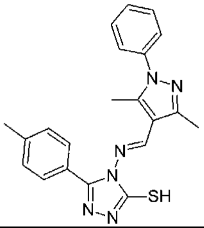	11	501	n.d.	n.d.	n.d.
83	22400643	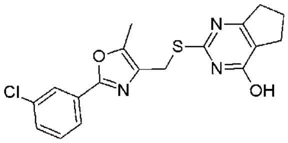	Single-ton	1259	n.d.	n.d.	n.d.
84	50106282	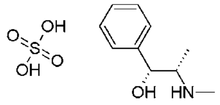	Single-ton	1413	0.7 ± 0.1	1.5 ± 0.2	-0.1 ± 0.2
85	26748001	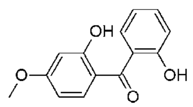	Single-ton	1000	n.d.	n.d.	n.d.
86	26748644	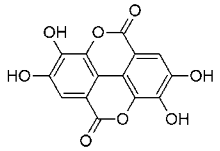	Single-ton	45	n.d.	n.d.	n.d.
87	22404741	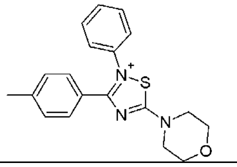	Single-ton	3162	N/A	N/A	N/A

* N/A, no unfolding transition observed in DSF; n.d., not determined
