# Supplementary material for: High-Affinity Inhibitors of Human NAD+-Dependent 15-Hydroxyprostaglandin Dehydrogenase: Mechanisms of Inhibition and Structure-Activity Relationships
Source: PLoS One. 2010 Nov 2;5(11):e13719. doi: 10.1371/journal.pone.0013719 (PMC2970562; doi:10.1371/journal.pone.0013719)
Supplement: Table S2 — Data collection and structural refinement statistics for human 15-PGDH. (0.11 MB PDF) [file pone.0013719.s002.pdf]

High-affinity Inhibitors of Human NAD$^+$-dependent 15-Hydroxyprostaglandin Dehydrogenase: Mechanisms of Inhibition and Structure-activity Relationships

Frank H. Niesen, Lena Schultz, Ajit Jadhav, Chitra Bhatia, Kunde Guo, David J Maloney, Ewa S. Pilka, Minghua Wang, Udo Oppermann, Tom D. Heightman and Anton Simeonov

SUPPLEMENTARY INFORMATION TABLE S2

Data collection and structural refinement statistics for human 15-PGDH.

**PDB code**	**2GDZ**
**Data Collection**	
X-ray source	*Rigaku FRE-Superbright*
Wavelength (Å)	*1.5418*
Space group	$P4_12_12$
Cell dimensions	
a, b, c (Å)	49.54, 49.54, 195.77
α, β, γ (°)	90.0, 90.0, 90.0
Resolution (Å)*	21.86 – 1.65 (1.74 – 1.65)
R_{merge}*	0.077 (0.688)
Mean(I/σI)*	23.9 (2.5)
Redundancy*	11.9 (7.2)
Completeness (%)*	99.6 (97.7)
**Refinement**	
Resolution (Å)	21.86 – 1.65
No. of reflections	28 883
R/R_{free}	17.7/ 21.2
No. of atoms	
Protein	2053
NAD	43
Water	303
R.m.s. deviations	
Bond lengths (Å)	0.019
Bond angles (°)	1.627

* Highest resolution shell shown in parentheses
