# Supplementary material for: High-Affinity Inhibitors of Human NAD+-Dependent 15-Hydroxyprostaglandin Dehydrogenase: Mechanisms of Inhibition and Structure-Activity Relationships
Source: PLoS One. 2010 Nov 2;5(11):e13719. doi: 10.1371/journal.pone.0013719 (PMC2970562; doi:10.1371/journal.pone.0013719)
Supplement: Figure S1 — Miniaturized screening assay for 15-PGDH. A. Activity heatmaps showing the assay miniaturization to a 4 µl volume in 1536-well format (increasing concentrations to the right). B. Plot of the Z' factor associated with each plate of the pilot concentration-response screen of the LOPAC1280 library performed to validate hit reproducibility. C. Molecular structure of the control inhibitor GW5074 that was added as a 16-point dilution series in duplicate between 57.5 µM and 1.75 nM into the second column of every assay plate (panel D). The average IC50 for the compound was 10.4 µM and the associated minimum significant ratio was approx. 1.5. E. Example of a fluorescent inhibitor (applied at between 3.5 nM and 57.5 µM) on the time course of NAD+-reduction upon injection of PGE2. (0.28 MB PDF) [file pone.0013719.s003.pdf]

High-affinity Inhibitors of Human NAD^+-dependent 15-Hydroxyprostaglandin Dehydrogenase: Mechanisms of Inhibition and Structure-activity Relationships

Frank H. Niesen, Lena Schultz, Ajit Jadhav, Chitra Bhatia, Kunde Guo, David J Maloney, Ewa S. Pilka, Minghua Wang, Udo Oppermann, Tom D. Heightman and Anton Simeonov

SUPPLEMENTARY INFORMATION FIGURE S1

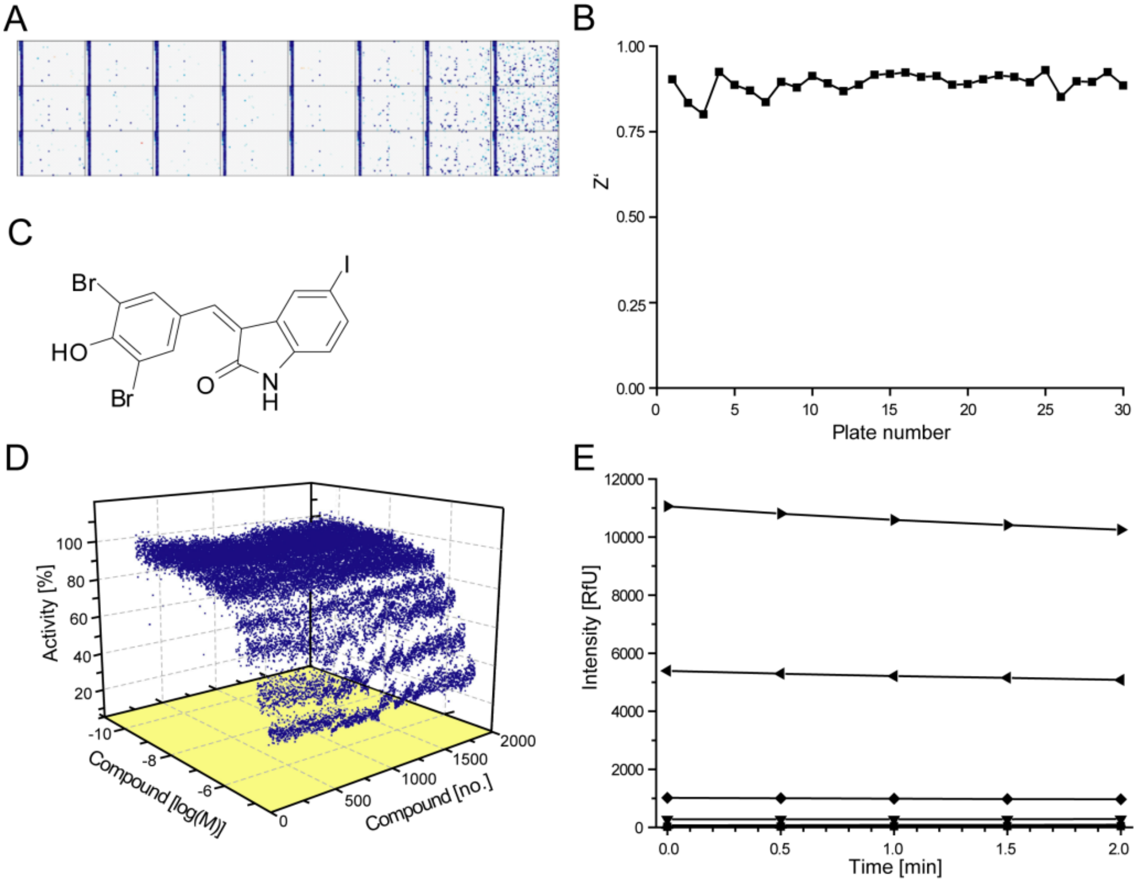

Miniaturized screening assay for 15-PGDH. *A.* Activity heatmaps showing the assay miniaturization to a 4 µl volume in 1536-well format (increasing concentrations to the right). *B.* Plot of the Z' factor associated with each plate of the pilot concentration-response screen of the $LOPAC^{1280}$ library performed to validate hit reproducibility. *C.* Molecular structure of the control inhibitor GW5074 that was added as a 16-point dilution series in duplicate between 57.5 µM and 1.75 nM into the second column of every assay plate (panel D). The average IC_{50} for the compound was 10.4 µM and the associated minimum significant ratio was approx. 1.5. *E.* Example of a fluorescent inhibitor (applied at between 3.5 nM and 57.5 µM) on the time course of NAD^+-reduction upon injection of PGE_2.
