# Supplementary material for: High-Affinity Inhibitors of Human NAD+-Dependent 15-Hydroxyprostaglandin Dehydrogenase: Mechanisms of Inhibition and Structure-Activity Relationships
Source: PLoS One. 2010 Nov 2;5(11):e13719. doi: 10.1371/journal.pone.0013719 (PMC2970562; doi:10.1371/journal.pone.0013719)
Supplement: Figure S2 — Binding of the cofactor NAD+ in the active site of human 15-PGDH. The acidic residue Asp36 forms hydrogen bonds to the 2′- and 3′-hydroxyl groups of the adenine ribose moiety, while Asp64 forms a hydrogen bond to the amino group of the adenine moiety. The nicotinamide moiety of NAD+ is positioned close to the catalytic tetrad (Asn107, Ser138, Tyr151 and Lys155). (0.08 MB PDF) [file pone.0013719.s004.pdf]

High-affinity Inhibitors of Human NAD$^+$-dependent 15-Hydroxyprostaglandin Dehydrogenase: Mechanisms of Inhibition and Structure-activity Relationships

Frank H. Niesen, Lena Schultz, Ajit Jadhav, Chitra Bhatia, Kunde Guo, David J Maloney, Ewa S. Pilka, Minghua Wang, Udo Oppermann, Tom D. Heightman and Anton Simeonov

SUPPLEMENTARY INFORMATION FIGURE S2

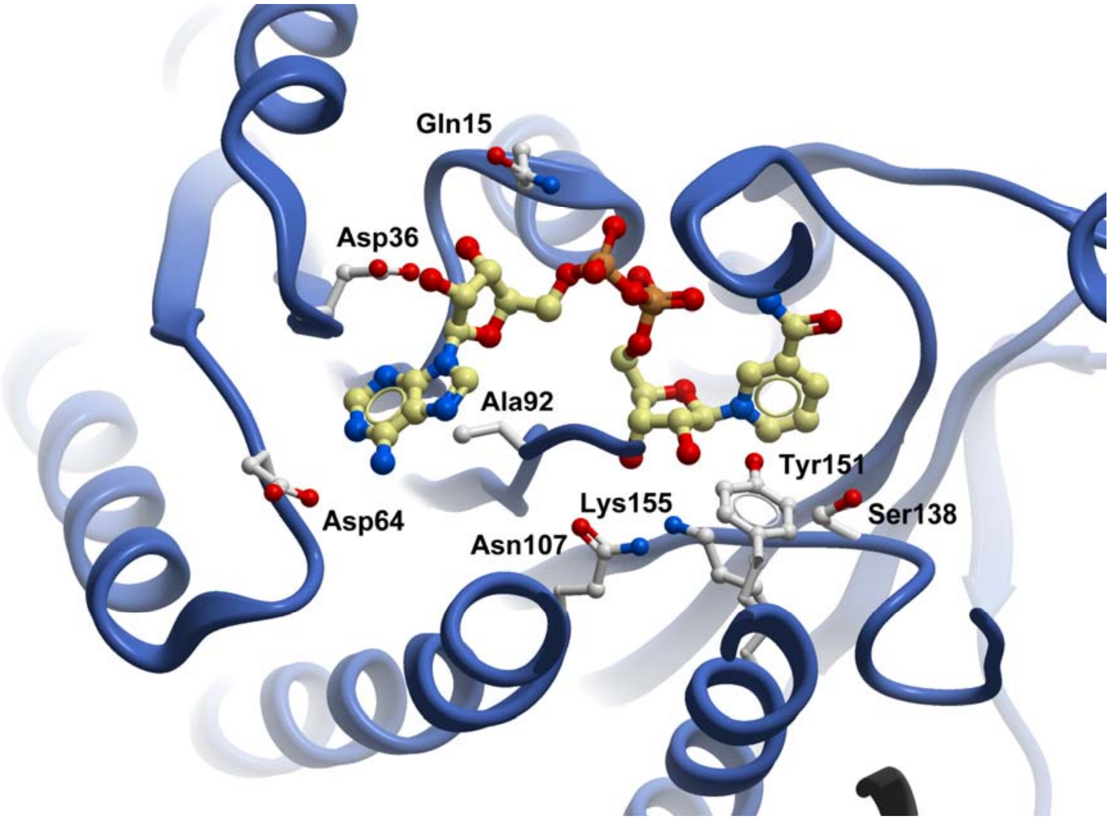

Binding of the cofactor NAD$^+$ in the active site of human 15-PGDH. The acidic residue Asp36 forms hydrogen bonds to the 2'- and 3'-hydroxyl groups of the adenine ribose moiety, while Asp64 forms a hydrogen bond to the amino group of the adenine moiety. The nicotinamide moiety of NAD$^+$ is positioned close to the catalytic tetrad (Asn107, Ser138, Tyr151 and Lys155).
