# Supplementary material for: High-Affinity Inhibitors of Human NAD+-Dependent 15-Hydroxyprostaglandin Dehydrogenase: Mechanisms of Inhibition and Structure-Activity Relationships
Source: PLoS One. 2010 Nov 2;5(11):e13719. doi: 10.1371/journal.pone.0013719 (PMC2970562; doi:10.1371/journal.pone.0013719)
Supplement: Figure S3 — Binding of inhibitor 72 in the active site of 15-PGDH as predicted by docking studies. The view shows a cut-through into the substrate pocket of 15 PGDH, with the volume of the pocket indicated by a green mesh. Key amino acid residues are labelled. The figures were created using ICM (Molsoft, LLC). (0.07 MB PDF) [file pone.0013719.s005.pdf]

# High-affinity Inhibitors of Human NAD$^+$-dependent 15-Hydroxyprostaglandin Dehydrogenase: Mechanisms of Inhibition and Structure-activity Relationships

Frank H. Niesen, Lena Schultz, Ajit Jadhav, Chitra Bhatia, Kunde Guo, David J Maloney, Ewa S. Pilka, Minghua Wang, Udo Oppermann, Tom D. Heightman and Anton Simeonov

## SUPPLEMENTARY INFORMATION FIGURE S3

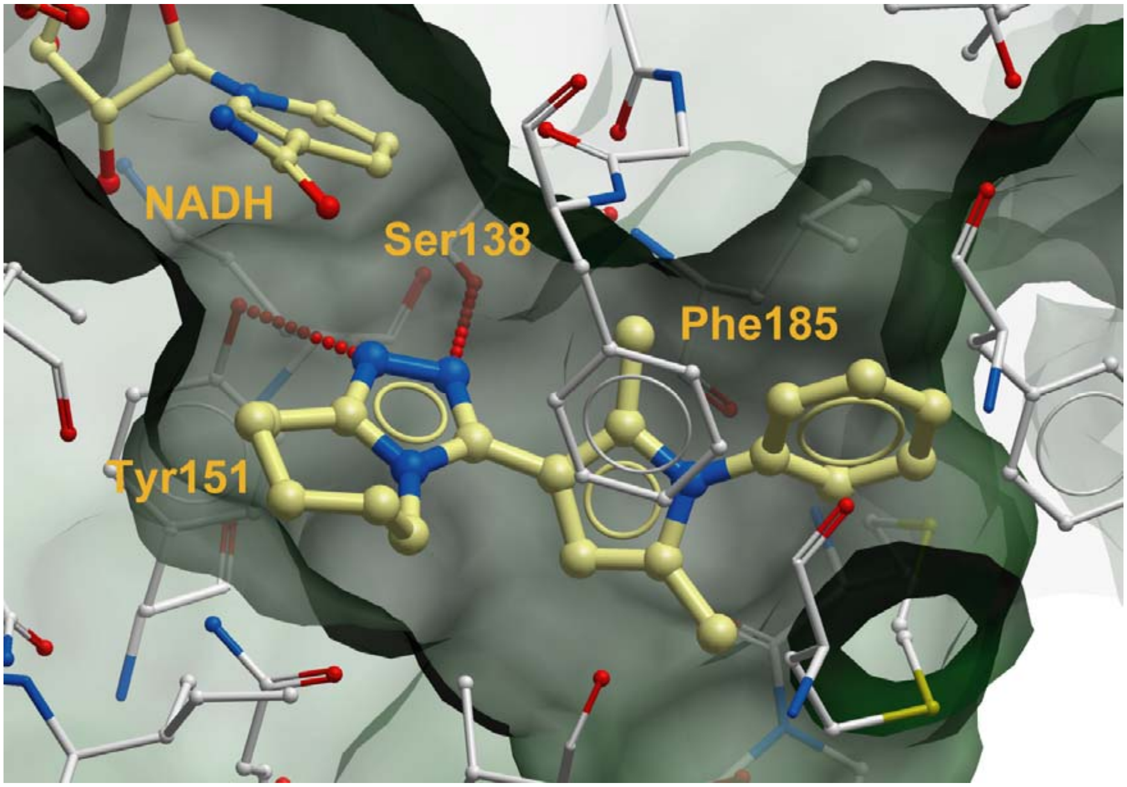


**Binding of the inhibitor 72 in the active site of 15-PGDH as predicted by docking studies.** The view shows a cut-through into the substrate pocket of 15-PGDH, with the volume of the pocket indicated by a green mesh. Key amino acid residues are labelled. The figures were created using ICM (Molsoft, LLC).
