# Supplementary material for: High-Affinity Inhibitors of Human NAD+-Dependent 15-Hydroxyprostaglandin Dehydrogenase: Mechanisms of Inhibition and Structure-Activity Relationships
Source: PLoS One. 2010 Nov 2;5(11):e13719. doi: 10.1371/journal.pone.0013719 (PMC2970562; doi:10.1371/journal.pone.0013719)
Supplement: Text S1 — Methods used in the chemical syntheses of the three lead compounds. (0.17 MB PDF) [file pone.0013719.s006.pdf]

High-affinity Inhibitors of Human NAD$^+$-dependent 15-Hydroxyprostaglandin Dehydrogenase: Mechanisms of Inhibition and Structure-activity Relationships

Frank H. Niesen, Lena Schultz, Ajit Jadhav, Chitra Bhatia, Kunde Guo, David J Maloney, Ewa S. Pilka, Minghua Wang, Udo Oppermann, Tom D. Heightman and Anton Simeonov

SUPPLEMENTARY INFORMATION TEXT S1

CHEMICAL SYNTHESIS

General Methods:

Unless otherwise stated, all reactions were carried out under an atmosphere of dry argon or nitrogen in dried glassware. Indicated reaction temperatures refer to those of the reaction bath, while room temperature (RT) is noted as 25 °C. All solvents were of anhydrous quality purchased from Aldrich Chemical Co. and used as received. Commercially available starting materials and reagents were purchased from Aldrich and were used as received.

Analytical thin layer chromatography (TLC) was performed with Sigma Aldrich TLC plates (5 x 20 cm, 60 Å, 250 µm). Visualization was accomplished by irradiation under a 254 nm UV lamp. Chromatography on silica gel was performed using forced flow (liquid) of the indicated solvent system on Biotage KP-Sil pre-packed cartridges and using the Biotage SP-1 automated chromatography system. ^{1}H- and ^{13}C-NMR spectra were recorded on a Varian Inova 400 MHz spectrometer. Chemical shifts are reported in ppm with the solvent resonance as the internal standard ($CDCl_3$ 7.26 ppm, 77.00 ppm, DMSO-d_6 2.49 ppm, 39.51 ppm for ^{1}H, ^{13}C respectively). Data are reported as follows: chemical shift, multiplicity (s = singlet, d = doublet, t = triplet, q = quartet, br = broad, m = multiplet), coupling constants, and number of protons. Low resolution mass spectra (electrospray ionization) were acquired on an Agilent Technologies 6130 quadrupole spectrometer coupled to the HPLC system. High resolution mass spectral data was collected in-house using an Agilent 6210 time-of-flight mass spectrometer, also coupled to an Agilent Technologies 1200 series HPLC system. If needed, products were purified via a Waters semi-preparative HPLC equipped with a Phenomenex Luna® C18 reverse phase (5 micron, 30 x 75 mm) column at a flow rate of 45 ml/min. The mobile phase was a mixture of acetonitrile and H_2O each containing 0.1% trifluoroacetic acid. Samples were analyzed for purity on an Agilent 1200 series LC/MS equipped with a Luna® C18 reverse phase (3 micron, 3 x 75 mm) column having a flow rate of 0.8-1.0 ml/min over both a 2.8-minute gradient/4.5 minute run time (short) and 6.8-minute gradient/8-minute run time (long). The mobile phase was a mixture of acetonitrile (0.025% TFA) and H_2O (0.05% TFA), and the temperature was maintained at 50 °C. Purity of final

compounds was determined to be >95%, using a 3 μl injection with quantitation by AUC at 220 and 254 nm (Agilent Diode Array Detector).

Chemical synthesis of compound **13**:

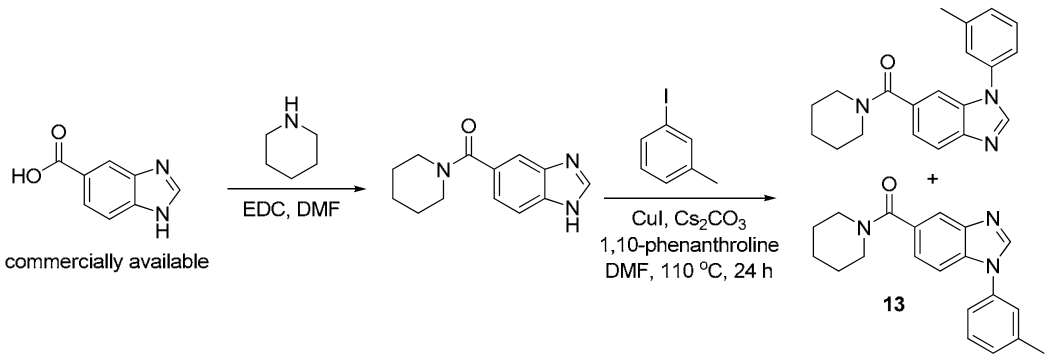

Scheme 1: Initial Synthetic route for the preparation of compound 13.

Initial attempts to synthesize compound **13** involved formation of the requisite amide using EDC coupling conditions with 5-benzimidazolecarboxylic acid and piperidine. Construction of the N-aryl linkage was achieved using a variety of conditions including using a Buchwald coupling with 1-iodo-3-methylbenzene in the presence of copper iodide, cesium carbonate and 1,10-phenanthroline in DMF at elevated temperatures (Scheme 1) [1]. Additionally, Suzuki-coupling conditions using the requisite boronic acid in a copper acetate-catalyzed reaction were attempted. Unfortunately, while both reactions gave the desired product to some proportion, the product ratio of the formed regioisomers was never greater than 1:1, and they could not be efficiently separated by either normal phase column chromatography or reversed-phase HPLC. As there was the additional concern that - even if the products could be separated - it may be difficult to unequivocally determine which is the desired regioisomer in the absence of an x-ray structure or advanced NMR techniques, we pursued an alternate route which, although it comprised a larger number of steps, left no doubt as to the proper structural assignment (Scheme 2).

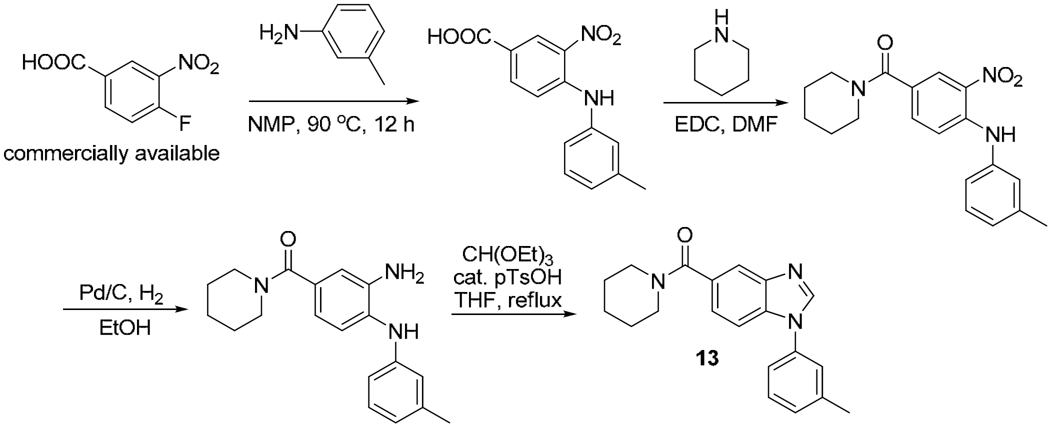

Scheme 2: Successful route to compound 13.

In this alternate route, the first step involved nucleophilic displacement of the aromatic fluoride on 4-fluoro-3-nitrobenzoic acid with 3-methylaniline using NMP as the solvent at 90 °C for 12 h. Formation of the amide proceeded smoothly using standard conditions (EDC, DMF) and subsequent reduction of the aromatic nitro group via hydrogenation with Pd/C, H_2 gave a high yield of the desired aromatic diamine. Finally, acid-catalyzed cyclization using triethylorthoformate in the presence of pTsOH in THF at reflux achieved the desired benzimidiazole product with a high yield [2].

Product characterization:

Piperidin-1-yl(1-m-tolyl-1H-benzo[d]imidazol-5-yl)methanone (**13**). ^{1}H NMR (400 MHz, DMSO-d_6) δ 1.3-1.65 (m, 6H), 2.43 (s, 3H), 3.47 (brs, 4H), 7.22-7.40 (m, 2H), 7.40-7.59 (m, 3H), 7.65 (d, 1H, J = 8.4 Hz), 7.75 (s, 1H) and 8.62 (s, 1H); LC/MS-retention time: (short) 3.147 min; (long) 4.604 min; HRMS: *mz* $(M+H)^+$ = 320.1768 (calculated for $C_{20}H_{22}N_30$ = 320.1763).

Chemical synthesis of compound **61**:

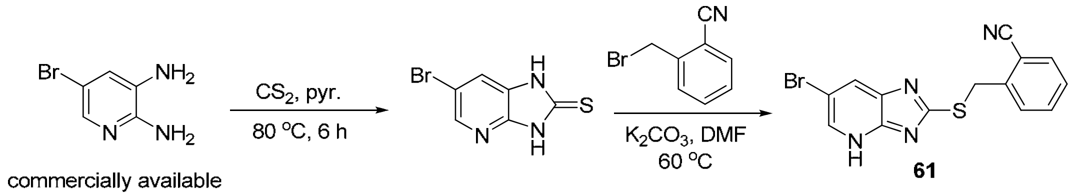

Scheme 3: Synthetic route for the preparation of compound 61

The synthesis of compound **61** was achieved in two steps from the commercially available 5-bromopyridine-2,3-diamine (Scheme 3). The first step involved cyclization to 6-bromo-1H-imidazo[4,5-b]pyridine-2(3H)-thione using carbon disulfide and pyridine as the solvent followed by acidification as described previously [3]. Alkylation of the sulfur moiety with the

requisite substituted benzyl bromide was accomplished using potassium carbonate in DMF at 60 °C to afford the desired molecule. Final product(s) were purified using reversed-phase preparative HPLC. Importantly, this functional route offers the possibility to synthesize additional analogues, by either changing the diamine or benzyl halide starting material.

Product characterization:

2-((6-bromo-4H-imidazo[4,5-b]pyridin-2-ylthio)methyl)benzonitrile (**61**). ^{1}H NMR (400 MHz, DMSO-d_6) δ 4.76 (s, 2H), 7.48 (t, 1H, J = 7.5 Hz), 7.66 (t, 1H, J = 7.6 Hz), 7.71 (m, 1H), 7.86 (d, 1H, J = 7.6 Hz), 8.13 (brs, 1H), 8.30 (d, 1H, J = 1.6 Hz) and 13.37 (brs, 1H); LC/MS-retention time: (short) 3.427 min; (long) 5.275 min; HRMS: *mz* $(M)^+$ = 343.9732 (calculated for $C_{14}H_9BrN_4S$ = 343.9731).

Chemical synthesis of compound **72**:

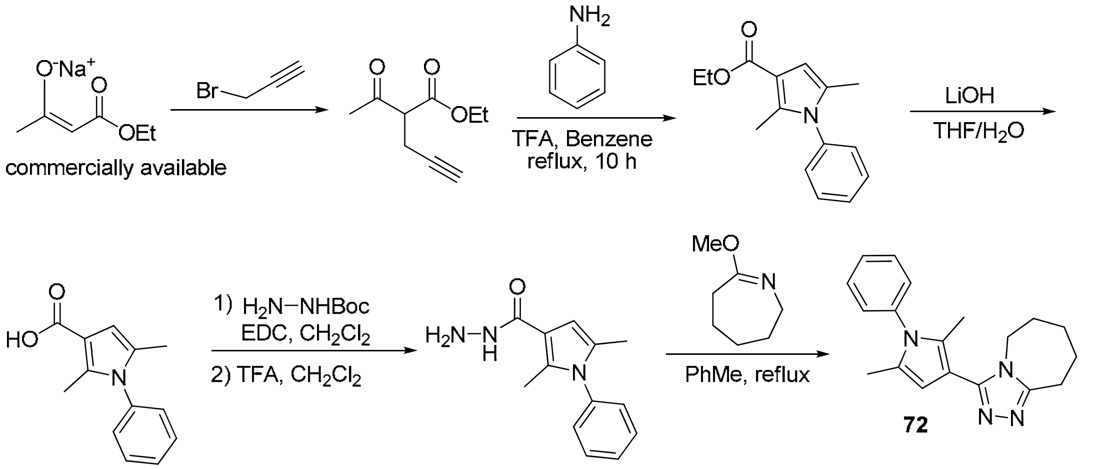

Scheme 4: Synthetic route for the preparation of compound 72.

The synthesis of compound **72** (Scheme 4) commenced with the preparation of ethyl 2-acetylpent-4-ynoate. Typical procedures involved treatment of ethyl acetoactate with sodium hydride followed by addition propargyl bromide [4,5], however, we chose to use the readily available sodium salt of ethyl acetoacetate which when reacted with propargyl bromide at room temperature in THF gave the desired product in high yield. Formation of the 1,2,3,5-tetra-substituted pyrrole was accomplished by treatment with aniline in the presence of 1 eq. of TFA in benzene at reflux which resulted in amination of the carbonyl, subsequent 5-exo-dig cyclization of the enaminone which was followed by aromatization [6]. Attempts to form the required hydrazine directly from the ethyl ester failed (hydrazine in ethanol at reflux for extended periods), so the ester was saponified using LiOH to provide the carboxylic acid

intermediate in good yield. Amidation with Boc-hydrazine using EDC-mediated coupling conditions followed by deprotection of the Boc group using TFA gave the hydrazide. Treatment of the acyl hydrazine with the commercially available imino ether in toluene at reflux gave the desired triazole in good yield [7].

Product characterization:

3-(2,5-dimethyl-1-phenyl-1H-pyrrol-3-yl)-6,7,8,9-tetrahydro-5H-[1,2,4]triazolo[4,3-a]azepine (**72**). ^{1}H NMR (400 MHz, DMSO-d_6) δ 1.62-1.68 (m, 4H), 1.80 (m, 2H), 2.01 (s, 6H), 2.91 (m, 2H), 4.01 (m, 2H), 6.02 (s, 1H), 7.36 (d, 2H, J = 7.4 Hz) and 7.44-7.60 (m, 3H); LC/MS-retention time: (short) 3.082 min; (long) 4.340 min; HRMS: *mz* $(M)^+$ = 306.1851 (calculated for $C_{19}H_{22}N_4$ = 306.1844).

REFERENCES

1. Antilla JC, Baskin JM, Barder TE, Buchwald SL (2004) Copper-diamine-catalyzed N-arylation of pyrroles, pyrazoles, indazoles, imidazoles, and triazoles. J Org Chem 69: 5578-5587.
2. (2007) Benzimidazole Derivatives and their use for modulating the $GABA_A$ receptor complex. NeuroSearch A/S. WO2007/65864 ed.
3. Yutilov MT, Scertilova IA (1988) Thionation of Imazopyridines. Chemistry of Heterocyclic Compounds 24: 653-658.
4. Trost BM, Rudd MT (2005) Ruthenium-catalyzed cycloisomerizations of diynols. J Am Chem Soc 127: 4763-4776.
5. Okuro K, Alper H (1996) Intramolecular and Intermolecular Mn(III)-Induced Carbon Monoxide Trapping Reactions of Alkynes with Malonate and Cyano Ester Units. Journal of Organic Chemistry 61: 5312-5315.
6. Demir AS, Aybey A, Kayalar M (2005) TFA catalyzed sequential amination/annulation/aromatization reaction of 2-proynyl-1,3-dicarbonyl compounds with amines. A new one-pot approach to functionalized pyrroles. ARKIVOC: 105-116.
7. Olson S, Aster SD, Brown K, Carbin L, Graham DW, et al. (2005) Adamantyl triazoles as selective inhibitors of 11beta-hydroxysteroid dehydrogenase type 1. Bioorg Med Chem Lett 15: 4359-4362.
